# Bacterial RNA downregulates MHC-I in tumor cell lines, promoting NK response and delaying tumor growth

**DOI:** 10.1371/journal.pone.0357999

**Published:** 2026-09-15

**Authors:** Agustina Serafino, Mónica Vermeulen, Yasmín A. Bertinat, Federico Birnberg-Weiss, Joselyn E. Castro, María Belén Bordignon, Ayelén I. Pesce Viglietti, Jorgelina Bueno, M. Ayelén Milillo, Paula Barrionuevo

**Affiliations:** 1 Instituto de Medicina Experimental-Consejo Nacional de Investigaciones Científicas y Técnicas (CONICET), Academia Nacional de Medicina, Buenos Aires, Argentina; 2 Centro de Investigaciones Oncológicas-Fundación Cáncer F.U.C.A, Buenos Aires, Argentina; 3 Instituto de Biociencias, Biotecnología y Biología traslacional. Facultad de Ciencias Exactas y Naturales. Universidad de Buenos Aires, Buenos Aires, Argentina; 4 Universidad Nacional de Río Negro. Instituto de Estudios en Ciencia, Tecnología, Cultura y Desarrollo. Consejo Nacional de Investigaciones Científicas y Técnicas, Río Negro, Argentina; The University of Texas MD Anderson Cancer Center, UNITED STATES OF AMERICA

## Abstract

Immunotherapy has introduced a new era in cancer treatment. The clinical goal of cancer immunotherapy is to prime the host immune system to provide passive or active immunity against malignant tumors. We have previously demonstrated that *Brucella abortus* (*Ba*) RNA downregulates IFN-γ-induced MHC-I surface expression in human monocytes/macrophages via a TLR8-dependent mechanism. Other bacterial RNAs can mimic this phenomenon. The presence and activity of NK cells in tumors have been correlated with better patient survival, supporting the evidence that these cells are essential in the immune response against tumors. So, we postulated that bacterial RNA (bacRNA) can be used to modulate MHC-I expression in tumors to enhance the NK cell response. Hence, the aim of this study was to investigate the immunomodulatory role of bacRNA in solid tumors. We first stimulated human glioblastomas U251 and LN-229, colorectal adenocarcinoma HT-29, breast cancer MCF-7, and murine melanoma B16-OVA cells with bacRNA in the presence of IFN-γ. Our experiments demonstrated that bacRNA diminished IFN-γ-induced MHC-I surface expression in all tumor cell lines. Moreover, the hTLR8 agonist ORN06/LyoVec mimicked the effect of bacRNA, indicating that MHC-I reduction would be mediated by TLR8. In addition, the decrease in MHC-I mediated by bacRNA correlated with increased NK cytotoxicity. Finally, treatment with either bacRNA or the ORN06 agonist resulted in greater immune cell infiltration and activation within the tumor compared to untreated mice. Furthermore, bacRNA delayed tumor growth in the B16 melanoma model. Overall, our established model of MHC-I downregulation (either by bacRNA or synthetic hTLR8 agonists) could be used as a therapeutic strategy to promote anti-tumor responses.

## Introduction

The interplay between tumor cells and their microenvironment is pivotal in regulating diverse hallmarks of tumor development, including its growth potential, its ability to invade tissues, and its capacity to generate metastases. This microenvironment consists of both immune cells and non-immune stromal cells, such as fibroblasts and endothelial [1]. However, the mechanisms of immune subversion that are characteristic of tumors constitutes an obstacle to the implementation of effective treatments [2]. The goal of cancer immunotherapy is to enhance the ability of the host immune system to eliminate malignant tumor cells [3]. Cancer cells escape immune surveillance by overexpressing checkpoint genes, which are crucial for the prevention of self-reactivity. In addition to well-known immune checkpoint genes, such as CTLA-4 and PD1, other genes have emerged as phagocytosis checkpoints. Genes such as CD47, CD24, MHC-I, PD-L1, STC-1, and GD2 function as “don’t eat me” signals or interact with “eat me” signals to suppress immune responses. Therefore, these checkpoints link the innate and adaptive immunity in cancer immunotherapy [4]. The field of immuno-oncology has experienced a paradigm shift with the advent of immune checkpoint inhibitors (ICIs). ICIs have been developed to interfere with the signaling transduction of inhibitory surface receptors present on immune cells, including both T lymphocytes and NK cells. Nevertheless, the fact that ICIs are associated with a poor response rate, numerous side effects, and the possibility that they may promote tumor exacerbation (although this has not yet been fully characterized) are significant concerning factors [2].

Natural killer (NK) cells are potent cytotoxic innate immune cells that play a fundamental role in tumor immunosurveillance by directly eliminating transformed cells and producing proinflammatory cytokines. However, in the immunosuppressive tumor microenvironment, NK cells become dysfunctional through exposure to inhibitory molecules produced by cancer cells, leading to tumor escape [5,6].

NK perform diverse roles across malignancies; for instance, robust NK cell infiltration is associated with prolonged survival in colorectal carcinoma, melanoma, and early-stage non-small cell lung cancer (NSCLC), whereas their dysfunction or decreased numbers are linked to disease progression in gastric, prostate, hepatocellular, and breast cancers. These anti-tumor responses are significantly influenced by pathogenic factors, particularly through the stimulation of Toll-like receptors (TLRs) by microbial-derived ligands [5,7].

For instance, the TLR5 agonist entolimod (former name CBLB502) rapidly activates TLR5-NF-kB signaling in hepatocytes and suppresses growth of both TLR5-expressing and non-expressing tumors in the liver through mobilization and activation of innate and adaptive immune mechanisms [8]. Similarly, priming with *Bacillus Calmette-Guérin* (BCG) can rescue dysfunctional NK cells from cancer patients, expanding a unique, long-lived CD56highCD16^+^ population that exerts potent cytotoxic activity against various solid tumors through NKG2D activation [6]. Furthermore, viral-derived factors, such as adenoviral CpG islands, can enhance anti-tumor immunity by stimulating TLR9 to increase NK cell-mediated killing of tumor cells [7]. These examples demonstrate how pathogen-associated molecular patterns could effectively modulate NK cell responses to overcome the immunosuppressive tumor microenvironment.

Recent research has demonstrated that the presence and activity of NK cells within tumors also enhanced patient survival outcomes [2]. Therefore, in this study, we aimed to investigate new strategies to awaken the immune system.

For several years, our lab has been investigating strategies employed by the intracellular pathogen *Brucella abortus* (*Ba*) to evade the immune response and persist chronically in the host. Specifically, we demonstrated that infection of monocytes/macrophages with *Ba* decreased IFN-γ-induced MHC-I surface expression, but only when *Ba* was alive. Consequently, *Ba*-infected macrophages display diminished capacity for antigen presentation to CD8^+^ T cells [9,10]. This led us to postulate that the *Ba* component(s) involved in the downregulation of MHC-I corresponded to a special class of viability-associated PAMPs known as *vita*-PAMPs, such as bacterial RNA [11,12], as they are found exclusively in living bacteria and are actively expressed during the early stages of infection. We also demonstrated that TLR8 is the receptor by which *Ba* RNA triggers a signal to downregulate MHC-I in human and murine macrophages [13,14]. Recently, we demonstrated that *Ba* RNA also reduces MHC-I expression in other cells that can be infected by bacteria, such as epithelial and endothelial cells [15]. Overall, our results describe a strategy through which *Ba* can avoid recognition by CD8^+^ T cells and evade immune surveillance. Moreover, our results demonstrate that other bacterial RNAs (such as the RNAs of *Salmonella typhimurium*, *Bacillus cereus*, *Escherichia coli* and *Klebsiella pneumonia*) can mimic this phenomenon [13]. Nevertheless, a downregulation in MHC-I surface expression leads not solely to diminished cytotoxic CD8^+^ T responses, but concomitantly to the triggering of NK cells [16]. Despite evidence that NK cells do not play a role in the defense against *Brucella* spp. infection [17], these cells are of pivotal significance within the antitumor defense mechanisms. Several bacteria have been shown to enhance immune responses and inhibit tumor progression [18–20]. Moreover, it has been reported that *Brucella* displays anti-tumor activity, and NK cells are postulated to be responsible for this activity [21].

These findings indicate the potential for utilization of bacterial RNA (bacRNA) in the modulation of MHC-I within tumors, where the NK cell response is of particular significance. In our lab, we are developing a model of MHC-I downregulation (either by bacRNA or synthetic hTLR8 agonists), which could be used as a therapeutic strategy to promote anti-tumor responses. Our *in vitro* experiments demonstrated that bacRNA diminished IFN-γ-induced MHC-I surface expression in several tumor cell lines. In addition, we demonstrated that the decrease in MHC-I mediated by bacRNA correlated with increased NK cytotoxicity. Furthermore, the results of this study demonstrated that treatment with either bacRNA or the ORN06 agonist resulted in greater immune cell infiltration and activation within the tumor compared to untreated mice. Furthermore, bacRNA delayed tumor growth in the B16 melanoma model. Here, we present the results of this study.

## Materials and methods

### Ethics statement

This study has been conducted according to the principles expressed in the Declaration of Helsinki. Peripheral blood mononuclear cells (PBMCs) were isolated exclusively from healthy adult donors according to the rules of the Ethics Committee of the Instituto de Medicina Experimental (IMEX)-Consejo Nacional de Investigaciones Científicas y Técnicas (CONICET), Academia Nacional de Medicina (protocol number 36/23/CEIANM corresponding to the original ethics approval document and 84/25/CEIANM corresponding to the ethics approval extension document). Donor samples were recruited in the period from July 6, 2023 to June 10, 2026. All donors provided a written informed consent before the study. The donations were anonymous and all donor information was protected. For the *in vivo* experiments, we used female C57BL/6 WT strain, 6–8 weeks-old. All procedures were approved and carried out under the conditions of the Institutional Committee on the Care and Use of Laboratory Animals (CICUAL: Comité Institucional para el Cuidado y Uso de Animales de Laboratorio) of the IMEX.

### Bacteria strains

*Brucella abortus* S2308, *Escherichia coli,* and *Klebsiella pneumoniae* were cultured in tryptic soy agar medium. The number of bacteria in the stationary-phase cultures was determined by comparing the optical density (OD) at 600 nm with a standard curve. All live *E. coli* and *K. pneumoniae* manipulations were performed in a biosafety level 2 laboratory (BSL-2) of the Instituto de Medicina Experimental (IMEX)-CONICET, Academia Nacional de Medicina. All live *Brucella* manipulations were performed in a BSL-3 of the Instituto de Investigaciones Biomédicas en Retrovirus y SIDA (INBIRS, UBA-CONICET), based on an agreement established in 2002.

### Cell cultures

For all experiments, human glioblastomas U251 and LN-229, colorectal adenocarcinoma HT-29 and HCT, breast cancer MCF-7, and murine melanoma B16-OVA cell lines were used. All experiments were performed at 37°C in a 5% CO_2_ atmosphere and a standard medium consisting of RPMI 1640 for B16-OVA cells or DMEM (Gibco) for U251, LN-229, HT-29, HCT, and MCF-7 cells, supplemented with 25 mM HEPES, 2 mM L-glutamine, 10% heat-inactivated FBS (Thermo Fisher Scientific), 100 U penicillin/ml, and 100 µg streptomycin/ml. U251, LN-229, HT-29, HCT, MCF-7, and B16-OVA cells were obtained from the American Type Culture Collection (Manassas, VA, USA) and were cultured as previously described. For cytotoxicity experiments or functional analysis, peripheral blood mononuclear cells (PBMCs) were obtained by centrifugation in Ficoll-Hypaque gradients (GE Healthcare) from human blood collected from healthy adult donors and resuspended in standard medium. PBMC effectors were thawed the evening before the assay and allowed to rest overnight (ON) in RPMI 1640 medium containing 10% FBS. For some experiments, NK cells were isolated from PBMC using the NK cell Isolation Kit (Miltenyi Biotec) following the manufacturer's instructions and allowed to rest ON. Cell viability was measured using the trypan blue exclusion test and was greater than 95% in all experiments. For all the experiments carried out, the cell confluence was 80%.

### RNA preparation

*Brucella abortus* S2308, *Escherichia coli* ATCC 25922 or *Klebsiella pneumoniae* ATCC 700603 (5 × 10^9^ CFU) were resuspended in 1 ml of TRIzol (Invitrogen), and RNA was purified using Quick-RNA DirectZol columns (Zymo Research), according to the manufacturer’s instructions. RNA quantification and purity were determined using a DeNovix DS-11 spectrophotometer (DeNovix Inc.). In all cases, the absorbance ratio of 260/280 nm was higher than 2.0, and the absorbance ratio of 260/230 nm was higher than 1.8. Equal amounts of RNA obtained from each bacterial species were then pooled to generate a single preparation, which was generically named bacRNA and used throughout the study.

### Viability assay

For viability assays, U251, LN-229, HT-29, HCT, MCF-7, and B16-OVA cell lines were treated with bacRNA or the TLR8 ligand ORN06 (InvivoGen) in the presence of IFN-γ (7.5 ng/ml) for 48 h. Cells were washed with PBS and resuspended in diluted 25 µL Zombie Violet™ solution to check the viability of the treated cells. Heat-killed cells were used as positive controls. The cells were incubated at room temperature in the dark for 15 min. Antibody surface staining was performed in the dark for 20 min. After labelling, the cells were analyzed using a Partec CyFlow (LabSystems) flow cytometer. Data were analyzed using FlowJo V10 software (FlowJo, LLC).

### *In vitro* stimulation

U251, LN-229, HT-29, HCT, MCF-7, and B16-OVA cells were incubated in 48-well plates (6 × 10^4^ cells/well) for 24 h to promote adherence. The cells were then stimulated with bacRNA or the TLR8 ligand ORN06 in the presence of IFN-γ for 48 h at 37°C in a 5% CO_2_ atmosphere. In all cases, the surface expression of MHC-I was evaluated using flow cytometry, as described in the following paragraph.

### Flow cytometry

U251, LN-229, HT-29, HCT, MCF-7, and B16-OVA cells were stained with anti-human MHC-I conjugated to FITC (W6/32) antibody or the corresponding isotype control antibody. After labelling, cells were analyzed on a FACSCalibur flow cytometer (BD Biosciences) or Partec CyFlow (LabSystems), and data were processed using FlowJo V10 software (FlowJo, LLC). Data were normalized to untreated cells, *i*.*e*., in each set of experiments, the MFI of treatments (from the % of positive cells for the marker) was divided by the MFI of the media condition (from the % of positive cells for each marker).

### Cytotoxicity assays

PBMC were normalized to the percentage of NK cells and used as effector cells, and different cancer cell lines were used as target cells. U251, LN-229, and HT-29 cells (used as target cells) were labelled with CFSE and incubated in 48-well plates (5 × 10^4^ cells/well) for 24 h to promote adherence. Then, the cells were stimulated with bacRNA (10 µg/ml) in the presence of IFN-γ for 48 h at 37°C in a 5% CO_2_ atmosphere. PBMC (used as effector cells) were co-cultured with CFSE-labelled tumor cells for 5 h. Co-cultures were performed at an E:T ratio of 3:1 (NK: target), following the method published by Esteso *et al*. [22] and Secchiari *et al*. [23]. The percentage of NK cells was determined before the experiment for each donor, so that equivalent effector NK cell-to-target ratios were set up for the different donors.

Cells were labelled with Zombie Violet™ solution to determine the viability of the treated tumor cells. Cytotoxicity was measured as the percentage of Zombie Violet-positive cells relative to the total number of CFSE-positive cells. After labelling, the cells were analyzed using a Partec CyFlow (LabSystems) flow cytometer. Data were analyzed using FlowJo V10 software (FlowJo, LLC).

In another set of experiments, U251 (target cells) was incubated in 48-well plates (5 × 10^4^ cells/well) for 24 h to promote adherence. Then, the cells were stimulated with bacRNA (10 µg/ml) in the presence of IFN-γ for 48 h at 37°C in a 5% CO_2_ atmosphere. Then, purified NK cells from healthy donors were co-cultured with the tumor cells in 96-well plates for 5 h. Co-cultures were performed at an E:T ratio of 3:1 (NK: target). The cells were labeled with CD56 and Zombie Violet™ solution to determine the viability of the treated tumor cells. U251 tumor cells were first gated based on FSC and SSC parameters, followed by selection of CD56-negative cells in an SSC *vs*. CD56 dot plot. Cytotoxicity was measured as the percentage of Zombie Violet-positive cells relative to the total number of CD56-negative U251 cells. After labelling, the cells were analyzed using a Partec CyFlow (LabSystems) flow cytometer. Data were analyzed using FlowJo V10 software (FlowJo, LLC).

### NK activation assay

U251 cells were incubated in 48-well plates (5 × 10^4^ cells/well) for 24 h to promote adherence. Then, the cells were stimulated with bacRNA (10 µg/ml) in the presence of IFN-γ for 48 h at 37°C in a 5% CO_2_ atmosphere. Purified NK cells were co-cultured with tumor cells in 96-well plates for 6 h at 37°C in 5% CO_2_ at an E:T ratio of 3:1 (NK: target), with the addition of FITC anti-human CD107a (clone H4A3, BD Pharmingen) from the beginning of the assay and the Protein Transport Inhibitor (Golgi Stop, BD) after the first hour. Cells were harvested, washed and labeled with PE-Cy5 anti-human CD56 (clone B159, BD Pharmingen) for 20 minutes at 4°C. The cells were then fixed with 1% PFA for 20 minutes and permeabilized using 0.5% saponin. Subsequently, the cells were labeled with PE anti-human IFN-γ (clone 4S.B3, BD Pharmingen) for 20 minutes at 4°C. After labelling, the cells were analyzed using a Partec CyFlow (LabSystems) flow cytometer. Data were analyzed using FlowJo V10 software (FlowJo, LLC). The results were expressed as the percentage of CD107a^+^ or IFN-γ^+^ cells after gating on CD56^bright^ NK cell subset.

### Injection of tumor cells into mice

The mice were placed in an isoflurane chamber for anesthesia. The B16-OVA melanoma cell suspension was then loaded into a 1 ml syringe with a 25G needle. The injection site was disinfected by spraying it with 70% ethanol. Finally, the needle was inserted subcutaneously and 100 μl of a 4 × 10^5^ B16-OVA melanoma cell suspension was slowly injected.

### Tumor monitoring and measurement

The tumors were measured when they became palpable. Tumor size was monitored every two days using a caliper. BacRNA (30 µg) or ORN06 (20 µg) or saline solution (vehicle) was injected intratumorally when the tumor reached a size of 5 mm × 5 mm. The tumor growth was monitored until the animals were euthanized. Tumor volume was calculated using the following formula: Tumor volume (mm³) = 4/3 x π x r^3^.

### Dissection of mice and dissociation of solid tumors

Mice were euthanized by CO_2_ inhalation followed by cervical dislocation. The mice were carefully dissected and solid tumors were collected. The harvested tumors were kept in PBS on ice, and preparations were made to proceed to the next step.

Tumor tissue was dissociated by pressing it against a 70 μm cell strainer using a syringe plunger. The resulting cell suspension was transferred to 15 ml centrifuge tubes and centrifuged at 400–600 × g for 5 minutes at 20–25°C. The supernatants were discarded by aspiration. The cells were washed with PBS and counted using the trypan blue dye.

### Identification of immune cells in solid tumors

After obtaining single-cell suspensions of solid melanoma tumors, the cells were stained with antibodies against surface molecules, using a standard protocol. To identify immune cell populations, cells were incubated with anti-mouse CD8 conjugated to PE (H35-17.2; BD Pharmingen), anti-mouse CD4 conjugated to FITC (GK1.5; BD Pharmingen), anti-mouse CD11c conjugated to PE (HL3; BD Pharmingen), anti-mouse CD11c conjugated to FITC (HL3; BD Pharmingen), anti-mouse CD11b conjugated to FITC (M1-70; BD Pharmingen), anti-mouse Ly6C conjugated to PerCP (HK1.4; BioLegend), anti-mouse NK-1.1 conjugated to APC (PK136; BioLegend), and anti-mouse NK-1.1 conjugated to PE (PK136; BioLegend) antibodies, or the corresponding isotype control antibodies. To determine the activation status of immune cells infiltrating the tumor microenvironment, cells were incubated with anti-mouse CXCR4 conjugated to PE/Dazzle (L276F12; BioLegend), anti-mouse I-A/I-E MHC-II conjugated to PE (M5/114.15.2; eBioscience), anti-mouse NKG2D conjugated to PE (1D11; BioLegend) and anti-mouse CD25 conjugated to PerCP-Cy5.5 (PCG1; BD Pharmingen) antibodies, or the corresponding isotype control antibodies. After labeling, the cells were analyzed using flow cytometry, as described in the corresponding paragraph.

### Measurement of extracellular IFN-γ

Single-cell suspensions were obtained from solid melanoma tumors, and 5 x 10^5^ cells were seeded in 96-well plates and incubated overnight. Subsequently, cells were collected and transferred to microcentrifuge tubes, centrifuged for 20 seconds, and the supernatants were carefully harvested. IFN-γ levels in the tumor cell supernatants were then determined by ELISA according to the manufacturer’s instructions (BD OptEIA Mouse IFN-γ ELISA Kit, Cat. No. 555138).

### Statistical analysis

Results were analyzed with one-way ANOVA followed by *post hoc* Tukey test or two-way ANOVA followed by *post hoc* Bonferroni test. Tumor growth curves were analyzed using two-way repeated-measures ANOVA with treatment (saline *vs.* bacRNA) and time as factors, followed by Sidak’s post hoc test for multiple comparisons between treatments at each time point. Statistical analyses were performed using GraphPad Prism 8.4.3 version. Statistical significance was set at *P* < 0.05.

## Results

### Bacterial RNA diminishes the IFN-γ-induced MHC-I surface expression on tumor cell lines and this is not due to a loss of cell viability

**Fig 1:**
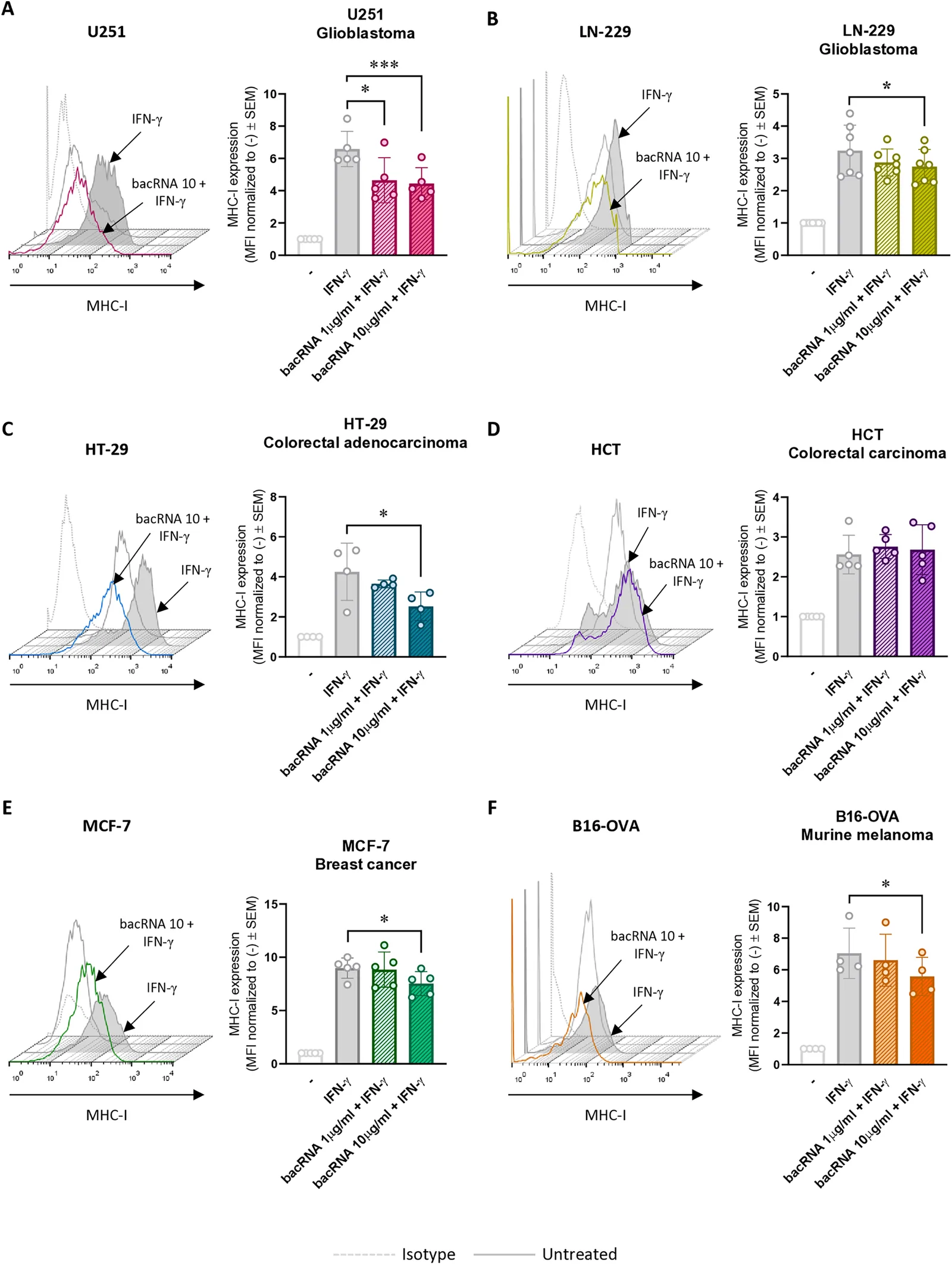
BacRNA diminishes the IFN-γ-induced expression of MHC-I in tumor cell lines. (A) U251, (B) LN-229, (C) HT-29, (D) HCT, (E) MCF-7, and (F) B16-OVA cell lines were attached to a 48-well plate for 24 h. Then, cells were stimulated with bacRNA (1 or 10 μg/ml) for 48 h with IFN-γ. MHC-I expression was determined by flow cytometry. Cells treated with IFN-γ were used as a positive control. Bars indicate the geometric mean ± SEM of at least four independent experiments. MFI, mean fluorescence intensity; (-), untreated cells; *P < 0.05; ***P < 0.001 *vs*. IFN-γ-treated cells.

To determine the effect of bacRNA on MHC-I expression, we performed experiments using different tumor cell lines: two human glioblastomas (U251 and LN-229), one colorectal adenocarcinoma (HT-29), one colorectal carcinoma (HCT), one breast cancer cell line (MCF-7), and an OVA-expressing murine melanoma (B16-OVA). Cells were stimulated with bacRNA (1 or 10 μg/ml) in the presence of IFN-γ for 48 h. MHC-I expression was measured using flow cytometry. The results showed that bacRNA diminished IFN-γ-induced surface expression of MHC-I in U251, LN-229, HT-29, MCF-7, and B16-OVA (Fig 1A-C, E, and F). However, MHC-I downregulation was not observed in the bacRNA-treated HCT cells (Fig 1D).

We then wondered whether the loss of cell viability was responsible for MHC-I downregulation in tumor cells. Consequently, we performed a viability assay using the Zombie violet staining method. Treatment with bacRNA did not significantly affect the viability of any of the cell lines evaluated (S1 Fig).

### An hTLR8 agonist mimics the effect of bacterial RNA on MHC-I surface expression

**Fig 2:**
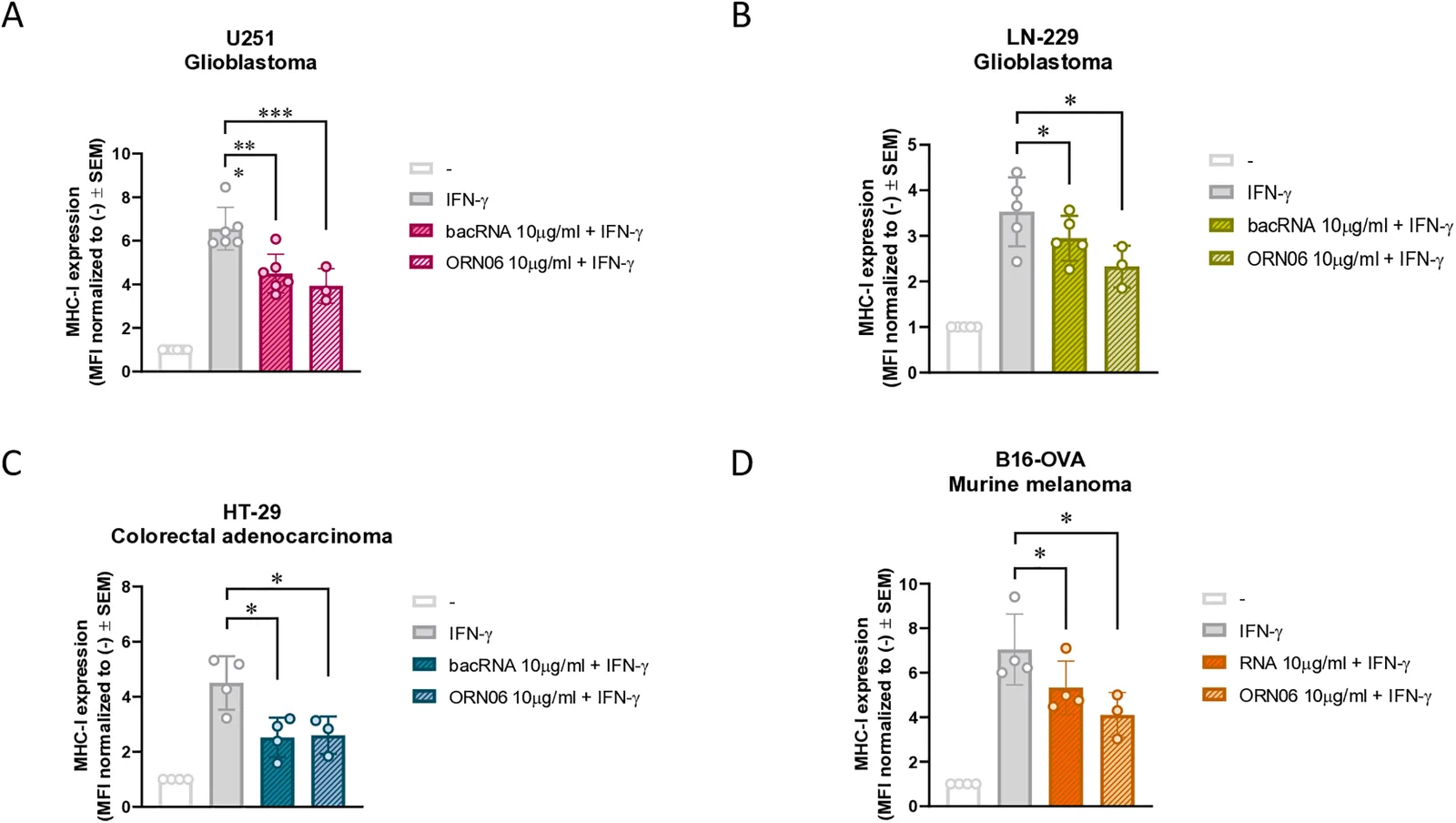
ORN06 mimics the effect of bacRNA on MHC-I expression. Different tumor cell lines were attached to a 48-well plate for 24 h. Then, cells were stimulated with ORN06 (10 μg/ml) for 48 h in the presence of IFN-γ. MHC-I expression in (A) U251, (B) LN-229, (C) HT-29, and (D) B16-OVA was determined by flow cytometry. Cells treated with IFN-γ were used as a positive control. Bars indicate geometric mean ± SEM of at least three experiments. MFI, mean fluorescence intensity; (-) untreated cells. *P < 0.05; ***P < 0.001 *vs*. IFN-γ-treated cells.

Our preliminary results indicated that *Ba* RNA downregulates IFN-γ-induced MHC-I surface expression in human monocytes/macrophages through a TLR8-dependent mechanism [10]. To delve deeper into these findings, U251, LN-229, HT-29 and B16-OVA cell lines were stimulated with 10 μg/ml of the synthetic TLR8 ligand ORN06 and IFN-γ for 48 h. MHC-I expression was evaluated by flow cytometry. ORN06 downregulated IFN-γ-induced MHC-I surface expression in all tumor cell lines evaluated (Fig 2A-D). Moreover, we showed that MHC-I downregulation mediated by ORN06 was not due to loss of cell viability (S2 Fig).

### The bacterial RNA-mediated MHC-I decrease correlates with an increased NK cytotoxicity

Next, we investigated whether the downregulation of MHC-I mediated by bacRNA could be a significant factor in the subsequent activation of NK cells against tumor cells.

**Fig 3:**
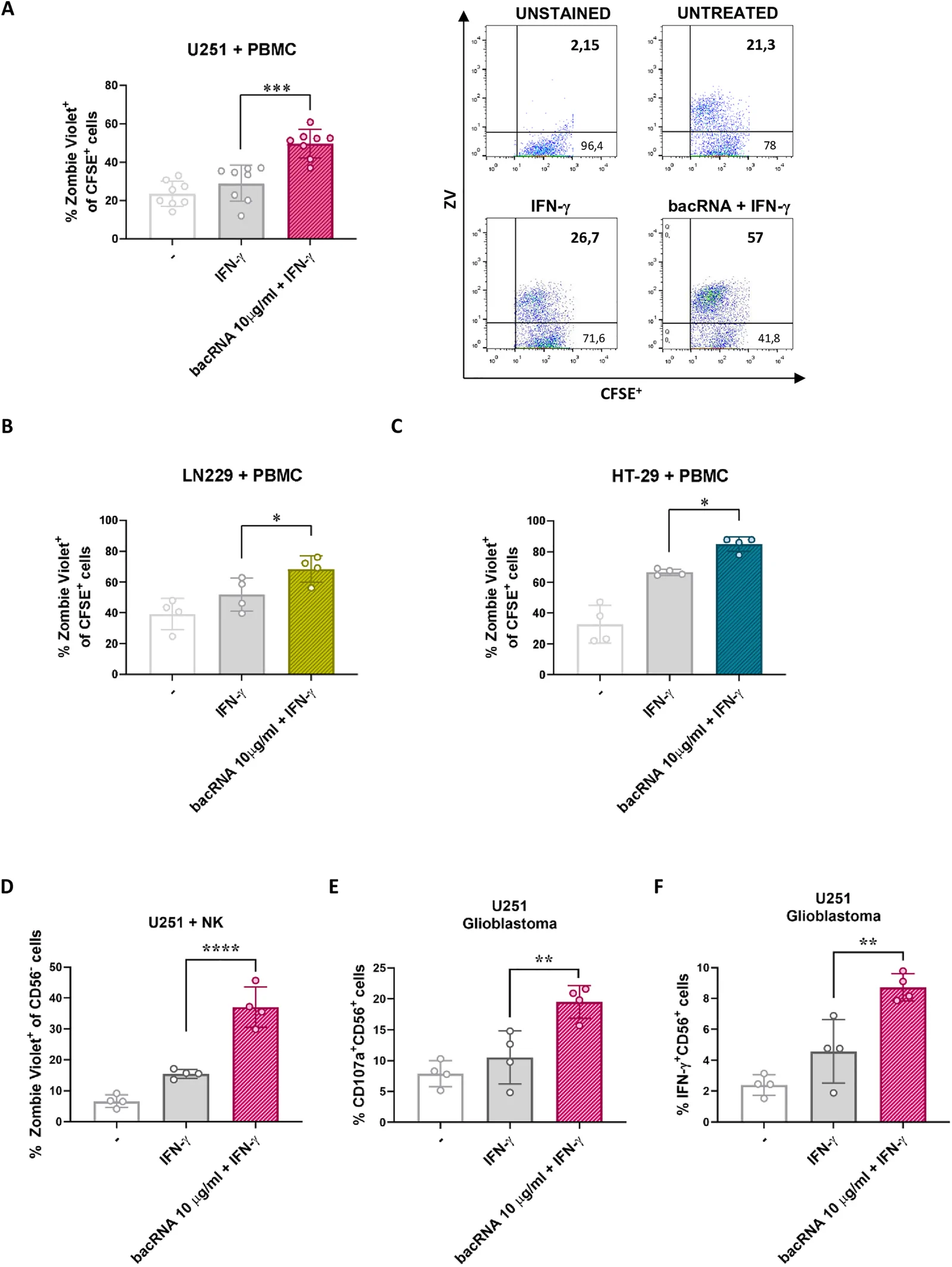
BacRNA-mediated MHC-I downregulation increases NK cytotoxicity and their activation against tumor cells. CFSE-pre-loaded (A) U251, (B) LN-229, and (C) HT-29 cell lines were attached to a 48-well plate for 24 h. Then, cells were stimulated with bacRNA (10 μg/ml) for 48 h, with IFN-γ. Afterward, target cells pre-loaded with CFSE were co-incubated with PBMCs (effector cells) for 5 h, and the cytotoxic response was evaluated with flow cytometry as the % of Zombie Violet (+) from CFSE (+) cells. In another set of experiments, U251 cells were attached to a 48-well plate for 24 h. Then, cells were stimulated with bacRNA (10 μg/ml) for 48 h, with IFN-γ. Afterward, target cells were co-incubated with purified NK cells (effector cells) for 5 h (for cytotoxicity) or 6 h (for activation). (D) Quantification of NK cytotoxicity as the % of Zombie Violet (+) from CD56 (-) cells. (E) Quantification of CD107a degranulation as the % of CD107a^+^CD56^+^ cells. (F) Quantification of IFN-γ production as the % of IFN-γ^+^CD56^+^ cells; (-) untreated cells. *P < 0.05; **P < 0.05; ***P < 0.001; ****P < 0.0001 *vs*. IFN-γ-treated cells.

For this purpose, we used CFSE-pre-loaded U251, LN-229, and HT-29 tumor lines as target cells. They were stimulated with bacRNA (10 μg/ml) and IFN-γ for 48 h. Additionally, PBMCs from healthy donors were purified and used as effector cells. Next, CFSE-pre-loaded target and effector cells were co-incubated for 5 h, and the cytotoxic response was evaluated by flow cytometry. Co-cultures were performed at an E:T ratio of 3:1 NK: target and the percentage of NK cells within PBMCs was determined before the experiment for each donor, so that equivalent effector NK cell-to-target ratios were set up for the different donors. We found that in all tumor cells evaluated, bacRNA increased cytotoxicity, as indicated by the percentage of Zombie Violet-positive from CFSE-positive cells (Fig 3A-C). To verify whether the observed cytotoxicity was indeed mediated by NK cells, we performed cytotoxicity assays using purified NK cells. For this, we stimulated U251 tumor line (as target cells) with bacRNA (10 μg/ml) and IFN-γ for 48 h. Additionally, NK cells from healthy donors were purified and used as effector cells. Next, target and effector cells were co-incubated for 5 h, and the NK cytotoxic response was evaluated by flow cytometry. Co-cultures were performed at an E:T ratio of 3:1 (NK: target). Our results demonstrated that in U251 tumor cells, bacRNA increased NK cytotoxicity, as indicated by the percentage of Zombie Violet-positive of CD56-negative cells (Fig 3D). In turn, our results showed that bacRNA increased the degranulation marker CD107a (Fig 3E) and IFN-γ production (Fig 3F) in NK cells (CD56^+^). Overall, these results indicate that bacRNA-mediated MHC-I downregulation correlates with an increase in NK cytotoxicity and their activation.

### Bacterial RNA promotes the infiltration of different immune cell populations and delays the tumor growth in a melanoma model

Given that downregulation of MHC-I by bacRNA may activate NK cells, we investigated the therapeutic potential of using bacRNA to modulate MHC-I as a strategy to stimulate an anti-tumor response.

**Fig 4:**
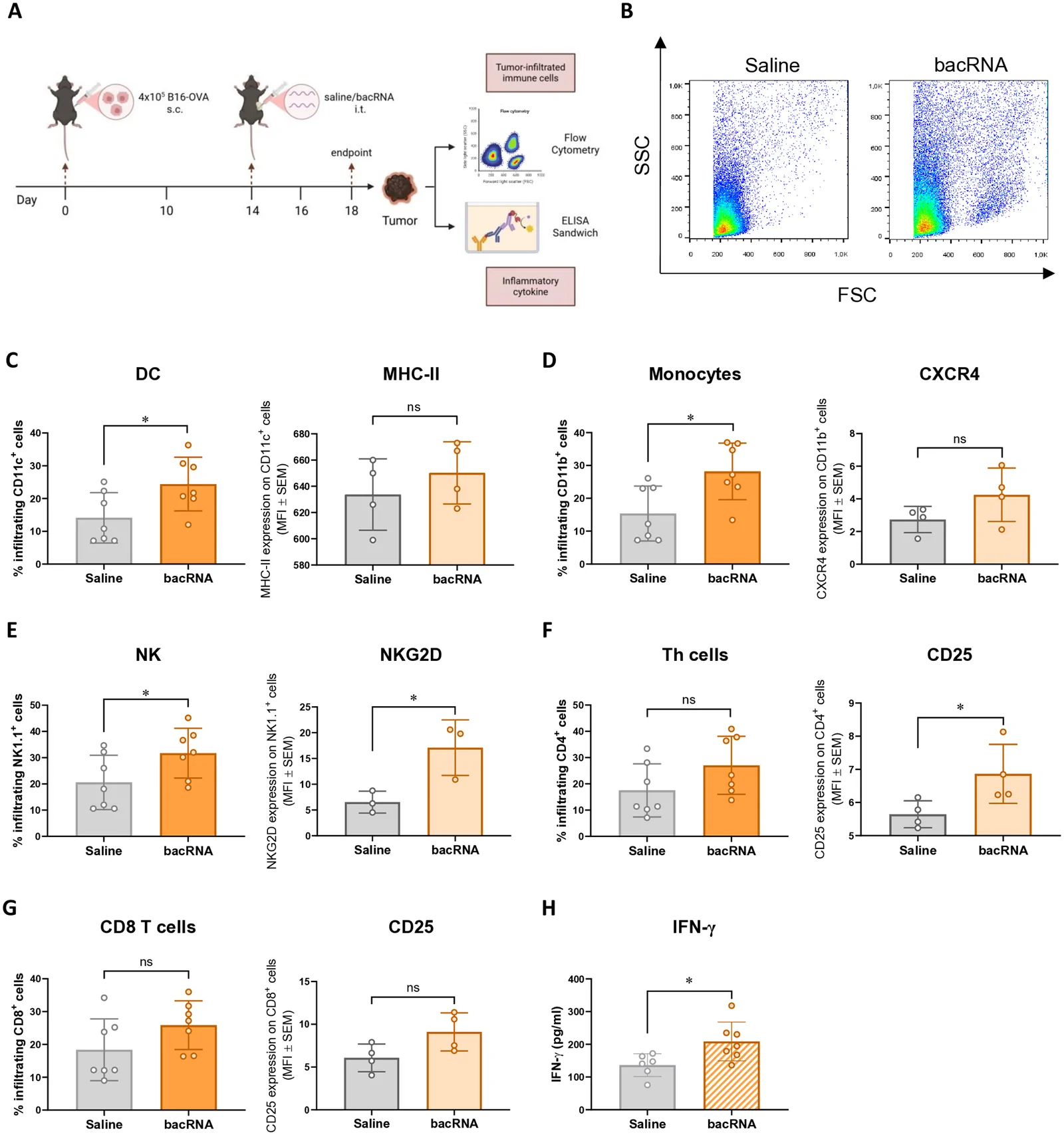
BacRNA promotes the infiltration of immune cells into the tumor. (A) Experimental design to characterize immune responses following bacRNA-based melanoma model treatment. Intratumoral bacRNA injections were administered after B16-OVA cell implantation (n = 7 per group). (B) Representative flow cytometry dot plot of the results shown in C-G. Detection of tumor infiltration and activation of dendritic cells (C), monocytes (D), NK cells (E), helper T cells (F) and CD8 T cells (G) by flow cytometry. Tumor cells were attached on a 96-well plate. After 24 h, supernatants were analyzed for the secretion of IFN-γ by sandwich ELISA (H). s.c.: subcutaneous injection; i.t.: intratumoral injection; ns, not statistically significant; *P < 0.05 *vs*. saline-treated tumor cells in C, D, E, F and H.

**Fig 5:**
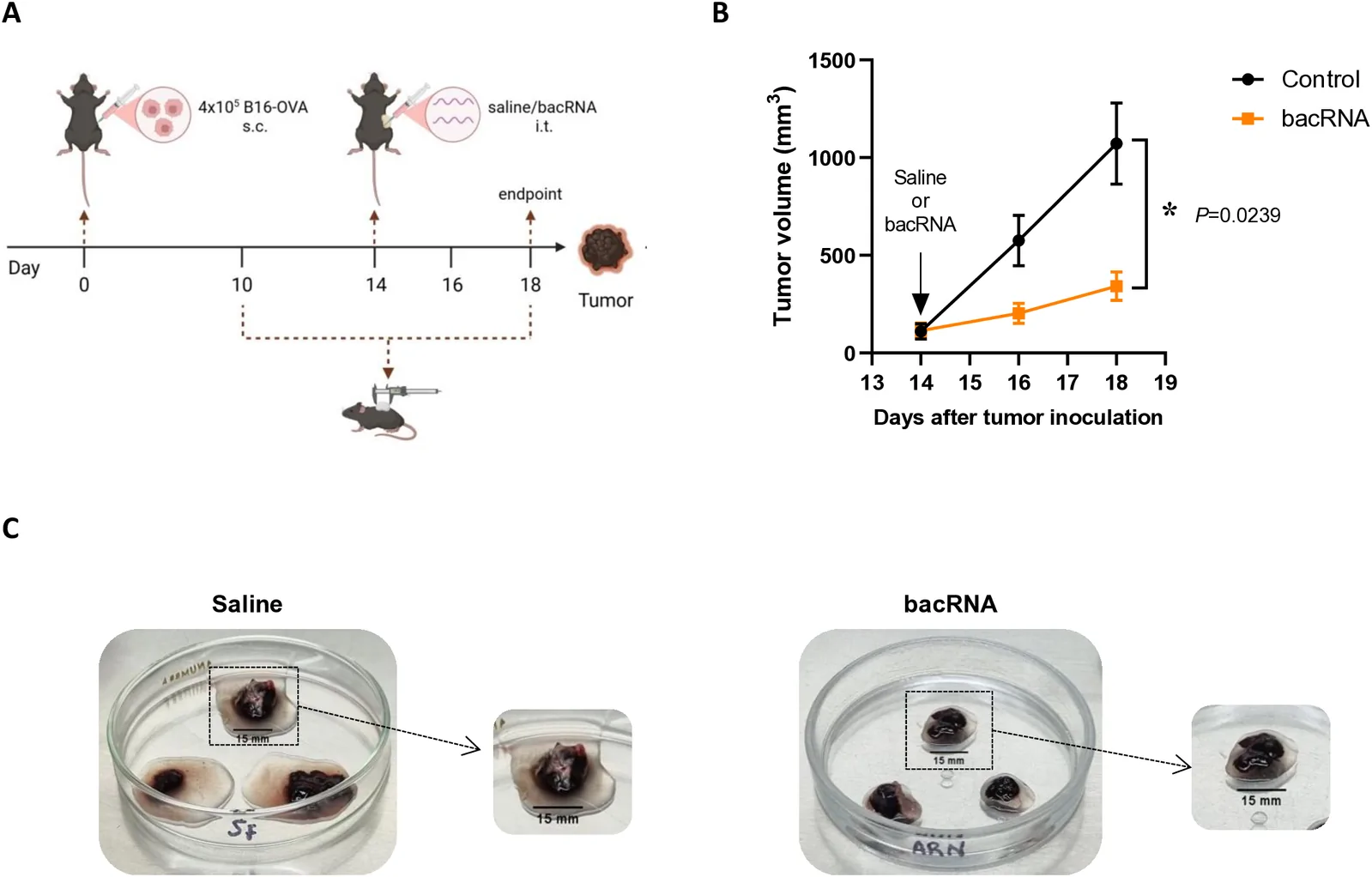
BacRNA delays tumor growth in the B16 melanoma model. (A) Experimental design to determine the size of the tumor at different time points post-treatment with bacRNA or saline solution. (B) Tumor growth curves of the intratumoral bacRNA treatment group *vs*. saline control group in the B16-OVA melanoma model, based on the experimental design in A. (C) Photos of tumors from the bacRNA treatment and control groups at the end of the experiment described in A. s.c.: subcutaneous injection; i.t.: intratumoral injection; Scale bar, 15 mm in C.

To achieve this, we evaluated the effect of bacterial RNAs on an *in vivo* tumor progression. Therefore, we used a murine melanoma model. Female C57BL/6 WT strain, 6–8 weeks-old, were inoculated subcutaneously with 4 × 10^5^ B16-OVA cells (n = 7 per group). When the tumor measured 5 mm × 5 mm, either bacRNA (30 μg) or saline solution was injected intratumorally. Next, we evaluated the size of the tumor at different post-treatment time points (Fig 5A). At the endpoint, we evaluated the infiltrating cells and their activation by flow cytometry, and the secretion of IFN-γ by ELISA (Fig 4A).

BacRNA promoted the infiltration of cells into the tumor microenvironment, as observed in the FSC *vs*. SSC dot plots (Fig 4B). Specifically, it increased the percentage of dendritic cells (DCs, CD11^+^ cells) and tended to increase MHC-II expression (Fig 4C). Also, bacRNA increased the percentage of monocytes (CD11b^+^ cells) and tended to increase CXCR4 expression (Fig 4D). BacRNA also increased the percentage of NK cells (NK1.1^+^ cells) and the expression of NKGD2 (Fig 4E). Moreover, bacRNA tended to increase the percentage of helper T cells (Th cells), which exhibited increased expression of CD25 (Fig 4F). Finally, we also observed that bacRNA tended to increase the percentage and activation of CD8 T cells (Fig 4G). In addition, bacRNA induced the secretion of IFN-γ as measured by ELISA in supernatants harvested from the tumor microenvironment (Fig 4H).

Additionally, the size of the tumors was measured at different post-treatment time points (Fig 5A). We observed that bacRNA-treated mice showed slower tumor progression than control mice (Fig 5B), as shown by the clear size reduction of the melanoma tumor at the endpoint (Fig 5C).

Overall, these results indicate that bacRNA treatment is associated with increased infiltration of different immune cell populations and elevated IFN-γ levels in the tumor microenvironment, which could delay tumor growth.

### An hTLR8 agonist mimics the effects of bacterial RNA in the B16 melanoma model

**Fig 6:**
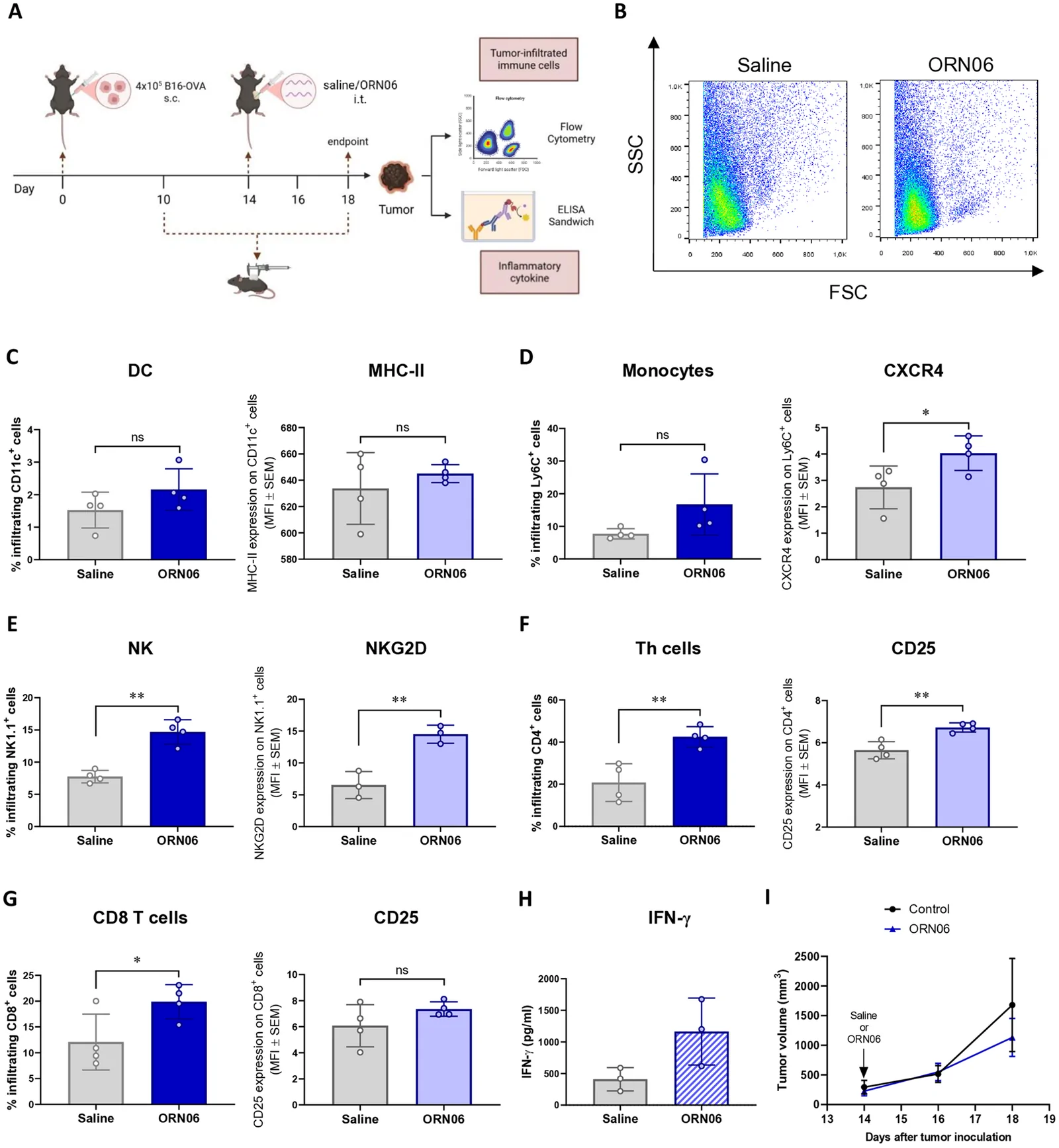
ORN06 mimics the effect of bacterial RNA in the B16 melanoma model. (A) Experimental design to characterize immune responses following ORN06-based melanoma model treatment. Intratumoral ORN06 injections were administered after B16-OVA cell implantation (n = 4 per group). (B) Representative flow cytometry dot plot of the results shown in C-G. Detection of tumor infiltration and activation of dendritic cells (C), monocytes (D), NK cells (E), helper T cells (F) and CD8 T cells (G) by flow cytometry. Tumor cells were attached on a 96-well plate. After 24 h, supernatants were analyzed for the secretion of IFN-γ by sandwich ELISA (H). (E) Tumor growth curves of the intratumoral ORN06 treatment group *vs*. saline control group in the B16-OVA melanoma model, based on the experimental design in A. s.c.: subcutaneous injection; i.t.: intratumoral injection; ns, not statistically significant; *P < 0.05 *vs*. saline-treated tumor cells in D (panel right) and G (panel left); **P < 0.01 *vs*. saline-treated tumor cells in E and F.

Taking into account that the TLR8 ligand ORN06, like bacRNA, is capable of down-regulating MHC-I surface expression in the B16-OVA tumor cell line (Fig 2D), we wondered whether this ligand could mimic the effects of bacRNA in the B16 melanoma model. For this, we performed the same *in vivo* experiments on tumor progression but injected the ORN06 agonist instead of bacRNA. Female C57BL/6 WT strain, 6–8 weeks-old, were inoculated subcutaneously with 4 × 10^5^ B16-OVA cells (n = 4 per group). When the tumor measured 5 mm × 5 mm, either ORN06 (20 μg) or saline solution was injected intratumorally. Next, we evaluated the size of the tumor at different post-treatment time points. At the endpoint, we evaluated the infiltrating cells and their activation by flow cytometry, and the secretion of IFN-γ by ELISA (Fig 6A).

ORN06 promoted the infiltration of cells into the tumor microenvironment, as observed in the FSC *vs*. SSC dot plots (Fig 6B), although to a lesser extent than bacRNA (Fig 4B). Specifically, ORN06 tended to increase the percentage and activation of DCs (Fig 6C). In turn, ORN06 tended to increase the percentage of monocytes, which exhibited increased expression of CXCR4 (Fig 6D). ORN06 increased the percentage of NK cells (NK1.1^+^ cells) and the expression of NKGD2 (Fig 6E). Moreover, ORN06 increased the percentage of Th cells and the expression of CD25 (Fig 6F). We also observed that ORN06 increased the percentage of CD8 T cells and tended to increase its activation marker (Fig 6G). In addition, ORN06 tended to induce the secretion of IFN-γ as measured by ELISA in supernatants harvested from the tumor microenvironment (Fig 6H). Finally, we observed that, at the endpoint, the tumor sizes of ORN06-treated mice were smaller than those of the control mice, although this difference was not statistically significant (Fig 6I).

Overall, these results indicate that ORN06 mimics the effects of bacterial RNA within the *in vivo* tumor microenvironment in the B16 melanoma model, but to a lesser extent than bacRNA.

## Discussion

Immunotherapy is a new focus of cancer research. It aims to strengthen or modify the host immune system in order to identify and destroy cancer cells more effectively. Recently, much attention has been paid to the potential use of bacteria in the development of novel cancer therapies [18,20]. Thus, bacteria may play opposing roles in immunotherapy [18,20]. First, they may participate in the formation, growth, spread, and resistance of tumors to chemotherapy [24–26]. These mechanisms include the stimulation of cancer cell proliferation by a pro-inflammatory microenvironment, changes in the tumor microenvironment due to bacterial metabolites, increased expression of oncogenic factors, immunosuppression, and initiation of the autophagy pathway to encourage chemotherapeutic resistance [27–30]. Conversely, some bacteria can enhance the immune response and inhibit tumor progression. This occurs in two main ways: bacteria can directly enhance the natural and specific responses of the immune system by increasing the expression of tumor antigens or by activating immune cells, or by indirectly regulating immune processes and modulating the tumor immune microenvironment by increasing cytokine and chemokine production [18]. Numerous bacteria can promote anti-tumor immunity through different mechanisms. Recently, it was demonstrated that intratissue bacteria from *Lachnospiraceae* family can promote immune surveillance by activating CD8^+^ T cells and controlling colorectal cancer progression [31]. *Lactobacillus* can enhance the innate immune response by activating pattern recognition receptors. They can stimulate dendritic cells (DCs) to secrete immune molecules, such as IFN-γ, which regulates the immune system and inhibits tumor growth [19]. Given the anti-tumor capacity of these bacteria, therapies based on their use are becoming a promising approach in cancer treatment because obligate or facultative anaerobic microorganisms may penetrate and proliferate in the hypoxic regions of tumors [32].

This study explored the potential of a bacterial *vita*-PAMP, its RNA, as a strategy to stimulate an anti-tumor response.

For years, our laboratory has been studying the strategies used by the intracellular bacterium *B. abortus* to evade the immune system and persist chronically in the host. We elucidated that the *vita*-PAMP RNA is a component employed by *B*. *abortus* to diminish MHC-I on monocytes/macrophages and other cells that can be infected by *B. abortus* [13–15]. Other bacterial RNAs can also mimic this phenomenon [13]. Given that a decrease in MHC-I can activate NK cells but that these cells do not play a role against *Brucella* spp. infection [17], we postulate that bacRNA could be used to downmodulate MHC-I levels in tumors for which the response of NK cells is crucial. Supporting our hypothesis, and in line with studies from other bacteria, it was reported that *Brucella* displays anti-tumor activity, with NK cells responsible for this effect [21]. However, the molecular mechanisms underlying this phenomenon have not been elucidated. In this study, we aimed to elucidate these mechanisms by exploring the ability of bacRNA to modulate MHC-I expression in several tumor cell lines. Our *in vitro* experiments demonstrated that bacRNA downregulates MHC-I on the surface of several human tumor cell lines (glioblastomas, colorectal adenocarcinoma, and breast cancer) and in a murine melanoma cell line. Moreover, we demonstrated that the decrease in MHC-I mediated by bacRNA correlated with increased NK cytotoxicity. These results could explain, at least in part, the previously reported anti-tumor activity of *Brucella* spp.

Our results also showed that the hTLR8 agonist ORN06 mimics the effect of bacRNA, indicating that MHC-I reduction would be mediated by TLR8. In line with this, evidence shows that TLR agonists are a promising class of anti-cancer agents that can effectively trigger immune responses in the tumor microenvironment [33]. Various TLR ligands have been widely used as adjuvants for the development of anti-cancer vaccines, some of which have been highly successful in clinical settings. The Food and Drug Administration (FDA) has approved three TLR agonists, *Bacillus Calmette-Guérin* (BCG; TLR4 agonist), monophosphoryl lipid A (MPLA; TLR2/4 agonist), and imiquimod (IMQ; TLR7 agonist), to treat bladder cancer, cervical cancer, and skin carcinoma, respectively [33]. In addition, there have been significant advances in the use of other TLR agonists such as oligonucleotides (ODN) containing unmethylated CpG sequences (TLR9 agonist) [34], short dsRNA (TLR3 agonist), and flagellin (TLR5 agonist) as vaccine adjuvants [33,35].

Agonists of TLR7/8 exhibit a promising capacity to function as vaccine adjuvants. These small molecules act directly on antigen-presenting cells (APCs), thereby amplifying immune responses, both humoral and cellular, with a particular emphasis on the Th1 type [36,37]. Both TLR7 and TLR8 recognize viral and bacterial single-stranded RNA [38–40]. The most widely studied synthetic TLR7/8 adjuvants are imidazoquinolines, including imiquimod (TLR7 agonist) and resiquimod (R848) (TLR7/8 agonist) [41,42]. Recently, it has been demonstrated that the TLR7/8 agonist R848 optimizes the host immune response in murine lung cancer [43]. Treatment with R848 resulted in increased levels of IFN-γ, TNF-α, and IL-2 as well as an increase in the proportion of DCs, NK, and CD8^+^ T cells, accompanied by a reduction in the proportion of Foxp3^+^ regulatory T cells within the tumor microenvironment [43]. In turn, it was demonstrated that the TLR7/8 agonist R848 induces anti-tumor responses and attenuates cachexia in murine models of pancreatic ductal adenocarcinoma [44]. It was also demonstrated that the combination of a TLR7/8 agonist and an epigenetic inhibitor increased the anti-tumor immune response against triple-negative breast cancer [45]. It has also been demonstrated that nanoparticles loaded with TLR7/8 agonists can be used as potent immunostimulatory adjuvants by promoting NK cell activation [46] and polarization of tumor-associated macrophages [47] to enhance cancer immunotherapy. At present, noteworthy focus is being directed towards the optimization of the composition and dissemination mechanisms employed by TLR7/8 agonists. The aim of that research is to optimize the retention of the adjuvants at the site of inoculation [36]. However, there have been few studies on the adjuvanticity of specific TLR8 agonists. Several years ago, it was demonstrated that the TLR8 agonist CL075 (also known as 3M-002) activates monocytes and myeloid DCs to induce robust cytokine production that drives adaptive immunity [48]. In contrast, the coordinated activation of TLR8 and NOD-like receptor pyrin domain containing 3 (NLRP3) inflammasome complex by the specific TLR8 agonist VTX-2337 triggers the tumoricidal activity of NK cells [49]. To date, the strongest evidence for the connection between TLR8-driven innate immune system activation by bacRNA and enhanced vaccine responses is observed in the context of live attenuated vaccines. The engagement of TLR8 on human monocytes/DCs by bacRNA derived from live *Escherichia coli* or the live attenuated vaccine BCG initiated Th1-polarised responses, directing Ag-specific CD4^+^ T cells in the generation of Tfh cells. This phenomenon was correlated with enhanced Ab-secreting plasmablasts and a more robust Ab response [38]. Consequently, the identification of TLR8 as a primary human sensor of *vita*-PAMPs and its role as a driver of Tfh cell differentiation renders TLR8 a promising target for Tfh cell–skewing vaccine adjuvants.

In line with the adjuvant capacity of TLR8 agonists, our *in vivo* experiments using a murine melanoma model showed that both bacRNA and the synthetic TLR8 agonist ORN06 promoted the infiltration and activation of multiple immune cell populations (DCs, monocytes, NK cells, Th cells, and CD8 T cells) into the tumor microenvironment. In addition, bacRNA treatment promoted increased IFN-γ secretion, consistent with its ability to enhance NK cell activation and cytotoxicity *in vitro*. These findings suggest that bacRNA, acting as a TLR8 agonist, not only enhances the NK cytotoxic response but also acts as an adjuvant. Taken together, these effects of bacRNA could contribute to the observed delay in tumor growth.

As previously stated, the utilization of bacteria as therapeutic agents in the management of tumors is a promising approach for cancer treatment [32]. Recent studies have suggested that TLR agonists, particularly TLR7/8 agonists, could be an effective way to reverse immunosuppression in the tumor microenvironment [33,43,44,46,47]. However, the use of bacRNA or the synthetic TLR8 agonist ORN06 as adjuvants in the development of anti-cancer vaccines has not yet been reported.

Overall, our *in vitro* and *in vivo* experiments show that bacRNA (or TLR8 agonists), alone or in combination with other TLR agonists or immunotherapies, could be a promising therapeutic strategy for promoting anti-tumor responses.

## Supporting information

S1 Fig BacRNA-mediated MHC-I downregulation is not due to a loss of cell viability. (A) U251, (B) LN-229, (C) HT-29, (D) HCT, (E) MCF-7, and (F) B16-OVA cell lines were attached to a 48-well plate for 24 h. Then, cells were stimulated with bacRNA (1 or 10 μg/ml) for 48 h with IFN-γ. Cell viability was evaluated with Zombie violet stain. Heat-killed cells were used as a positive control; (-) untreated cells.
(TIF)

S2 Fig ORN06-mediated MHC-I downregulation is not due to a loss of cell viability. (A) U251, (B) LN-229, (C) HT-29 and (D) B16-OVA cell lines were attached to a 48-well plate for 24 h. Then, cells were stimulated with ORN06 (10 μg/ml) for 48 h with IFN-γ. Cell viability was evaluated with Zombie violet stain. Heat-killed cells were used as a positive control; (-) untreated cells.
(TIF)
