# Supplementary material for: Bacterial RNA downregulates MHC-I in tumor cell lines, promoting NK response and delaying tumor growth
Source: PLoS One. 2026 Sep 15;21(9):e0357999. doi: 10.1371/journal.pone.0357999 (PMC13577469; doi:10.1371/journal.pone.0357999)
Supplement: Supplementary file 1 [file pone.0357999.s004.docx]

**Sriram Chitta**

Academic editor

*PLOS One*,

July, 14^th^ 2026

Dear Dr.,

Enclosed please find the revised version of the manuscript (PONE-D-26-11444) entitled “Bacterial RNA downregulates MHC-I in tumor cell lines, promoting NK response and delaying tumor growth” written by Agustina Serafino, Mónica Vermeulen, Yasmín A. Bertinat, Federico Birnberg-Weiss, Joselyn E. Castro, M. Belén Bordignon, Ayelén I. Pesce Viglietti, Jorgelina Bueno, M. Ayelén Milillo and Paula Barrionuevo. I am also sending the manuscript with highlighted changes as requested.

We would like to thank you and the two reviewers for their critiques, which have been very helpful in improving the quality of our manuscript. According to the guidelines for revised manuscripts, a letter in which a point-by-point response to your comments and those of the reviewers has been included below. Modifications of the manuscript’s text have been added to the revised version marked with yellow highlighting to indicate the changes.

The manuscript has not been submitted for publication or accepted elsewhere and all authors have seen and approved the content and have contributed significantly to the work. The authors declare no conflict of interest.

Hoping that you will find the revised version of our article suitable for publication in *PLOS One*, we thank you very much for the consideration you are giving to our manuscript.

Sincerely yours,

Paula Barrionuevo, Ph.D.

On behalf of all authors

**Comments:**

**Academic Editor (AE):**

**1.** Authors should show MHC I down regulation by bRNA in graded concentrations, by selecting one low (no inhibition) and one high (max inhibition). The data showed concentrations in plateau region.

As suggested by the AE, we now show MHC-I down-regulation by bacRNA in one low (1 μg/ml) and one high (10 μg/ml) concentration (Figure 1 of the revised version of the manuscript).

**2.** MFI/SEM values on Y axis of fig 1 & 3: Can you show a representative GM value for isotype, MHC-1 in presence and absence of bRNA. The value showed appears as "fold" change.

As mentioned by the AE, the data were presented as a “fold” change to minimize dispersion among the data obtained in the independent experiments. However, as suggested by the AE, we present the mean fluorescence intensity (MFI) values corresponding to all the graphs in Figure 1 and Figure 2 (which was Figure 3 in the original version of the manuscript). To organize these values, we designed the tables shown below. Given that raw data has been uploaded to the Zenodo public repository (<https://doi.org/10.5281/zenodo.21107026>), we decided not to add these tables onto the final version of the manuscript.

| **Figure 1** | **RAW DATA** | | | |
| --- | --- | --- | --- | --- |
|  | **-** | **IFN-γ** | **bacRNA 1 + IFN-γ** | **bacRNA 10 + IFN-γ** |
| U251 | 16.70 | 75.1 | 59 | 48.5 |
|  | 22.10 | 132 | 82.96 | 78.2 |
|  | 18.5 | 109.95 | 89.17 | 76.25 |
|  | 20.3 | 119.83 | 83.88 | 80.13 |
|  | 19.6 | 165.93 | 136.98 | 119.16 |
| LN-229 | 58.4 | 256.51 | 176.99 | 208.22 |
|  | 62.2 | 204 | 194 | 182 |
|  | 76.4 | 186 | 176 | 173 |
|  | 33.9 | 124 | 90.3 | 95 |
|  | 17.2 | 44.2 | 44.8 | 37.6 |
|  | 40 | 99.1 | 112.74 | 92.6 |
|  | 55 | 218.9 | 197.62 | 179.25 |
| HT-29 | 94.9 | 406 | 341 | 304 |
|  | 79.7 | 177 | 276 | 126 |
|  | 35.5 | 189 | 139 | 104 |
|  | 32.8 | 170 | 119 | 78.4 |
| HCT | 51 | 116 | 133 | 119 |
|  | 47 | 107 | 113 | 119 |
|  | 43.2 | 99.7 | 115 | 82.3 |
|  | 59.9 | 152 | 190 | 196 |
|  | 50.2 | 171 | 148 | 169 |
| MCF-7 | 16.7 | 124 | 119 | 104 |
|  | 16.2 | 146 | 119 | 106 |
|  | 14.8 | 135 | 164.72 | 118.71 |
|  | 16.5 | 165 | 156.99 | 147.5 |
|  | 12.2 | 114 | 111 | 96.5 |
| B16-OVA | 13 | 122.4 | 117 | 92.4 |
|  | 19.86 | 130 | 125 | 118.6 |
|  | 16.4 | 101.99 | 86.99 | 73.5 |
|  | 17.9 | 107.52 | 104.53 | 85.12 |

| **Figure 2** | **RAW DATA** | | | |
| --- | --- | --- | --- | --- |
|  | **-** | **IFN-γ** | **bacRNA 10 + IFN-γ** | **ORN06 + IFN-γ** |
| U251 | 16.70 | 75.1 | 48.5 |  |
|  | 22.10 | 132 | 78.2 |  |
|  | 18.5 | 109.95 | 76.25 |  |
|  | 20.3 | 119.83 | 80.13 | 66.505 |
|  | 19.6 | 165.93 | 119.16 | 94.325 |
|  | 21.8 | 139.72 | 104.2 | 80.78 |
| LN-229 | 76.4 | 186 | 173 | 141.378 |
|  | 58.4 | 256.51 | 208.22 | 161.4 |
|  | 62.2 | 204 | 182 | 147.15 |
|  | 33.9 | 124 | 95 |  |
|  | 55 | 218.9 | 179.25 |  |
| HT-29 | 94.9 | 406 | 304 | 297.43 |
|  | 87.4 | 281.5 | 138.17 | 160.48 |
|  | 35.5 | 189 | 104 | 100.66 |
|  | 32.8 | 170 | 78.4 |  |
| B16-OVA | 13 | 122.4 | 92.4 | 55.79 |
|  | 19.86 | 130 | 118.6 | 59.99 |
|  | 16.4 | 101.99 | 73.5 | 82.1148 |
|  | 17.9 | 107.52 | 85.12 |  |

**3.** Viability figs can go as Supplement figs as they are not adding any additional information

As suggested by the AE, the cell viability figures (Figure 2 and Panels D, E and F of Figure 3 in the original version of the manuscript) have been moved to the Supporting information section (Figure S1 and Figure S2 of the revised version of the manuscript).

**4.** (Same as reviewer 2): Since the main point of manuscript deals with NK cell activity, the experiment shown in manuscript is not In-depth one. When PBMC were used as effector cells, percentage of NK cells will be very low in numbers. it is advised to use PBMC in graded doses, to measure killing activity. Authors are advised to re-run this experiment, as suggested.

Since our manuscript focuses on the cytotoxic activity of NK cells, we agree with the AE and the reviewer 2 on the importance of strengthening the cytotoxicity results obtained using whole PBMCs (corresponding to Figure 4 in the original version of the manuscript). For this reason, we have performed cytotoxicity experiments with purified NK cells using the U251 tumor line. NK cells were isolated from PBMC using the NK cell Isolation Kit (Miltenyi Biotec). Our cytotoxicity experiments with purified NK cells replicated the results we had reported in the original version of the manuscript using whole PBMCs. These results confirm, at least for the U251 tumor line, that treatment with bacRNA effectively enhances the cytotoxicity of NK cells. These results have been incorporated as Panel D in Figure 3 of the revised version of the manuscript. Furthermore, as suggested by the reviewer 2, we performed NK cell activation experiments using the U251 tumor line. In these assays, we evaluated both CD107a degranulation and IFN-γ production. Our results showed that not only does treatment with bacRNA increase the cytotoxic capacity of NK cells, but also activate them, as evidenced by greater CD107a degranulation and an increased IFN-γ production. These results have been incorporated as panels E and F in Figure 3 of the revised version of the manuscript. We have added a paragraph describing these results in the Results section (Page 16, lines 361–370 of the revised version of the manuscript). Additionally, we expanded the “Cell cultures” paragraph [Page 7 (lines 163-165) and page 8 (line 166) of the revised version of the manuscript], the “Cytotoxicity assays” paragraph (Page 10, lines 221-230 of the revised version of the manuscript) and wrote a new “NK activation assay” paragraph in the Materials and Methods section [Page 10 (lines 232–236) and page 11 (237-245) of the revised version of the manuscript]. Moreover, we have extended the legend of Figure 3 (Page 17, lines 379-385 of the revised version of the manuscript).

The cytotoxicity experiments with purified NK cells were conducted in collaboration with María Belén Bordignon and Ayelén Ivana Pesce Viglietti from “Centro de Investigaciones Oncológicas-Fundación Cáncer FUCA (Buenos Aires, Argentina)”. For this reason, they were added as authors to the revised version of the manuscript.

**5.** All immune cells (CD4, DC, Monocytes, CD8 T cells (authors wrongly used as CTL?) increased with bRNA injection in tumors. There is no specific increase in NK cells alone. Can authors comment on?

We agree with the AE that the increase not only in NK cells but also in other immune cells is a very important point that must be taken into account when interpreting and discussing the results. In fact, in the discussion section of the original manuscript, we dedicated a lengthy paragraph to the role of TLR 7/8 agonists as vaccine adjuvants in different tumor contexts [Page 23 (lines 527-545) and page 24 (lines 546-560) of the revised version of the manuscript]. Some of the cited studies mention: 1) Treatment with R848 resulted in increased levels of IFN-γ, TNF-α, and IL-2 as well as an increase in the proportion of DCs, NK, and CD8^+^ T cells, accompanied by a reduction in the proportion of Foxp3^+^ regulatory T cells within the tumor microenvironment [43] and 2) It has also been demonstrated that nanoparticles loaded with TLR7/8 agonists can be used as potent immunostimulatory adjuvants by promoting NK cell activation [46] and polarization of tumor-associated macrophages [47] to enhance cancer immunotherapy.

Furthermore, in our discussion, we explain the infiltration of various cell populations into the tumor microenvironment as follows:

“In line with the adjuvant capacity of TLR8 agonists, our in vivo experiments using a murine melanoma model showed that both bacRNA and the synthetic TLR8 agonist ORN06 promoted the infiltration and activation of multiple immune cell populations (DCs, monocytes, NK cells, Th cells, and CD8 T cells) into the tumor microenvironment. In addition, bacRNA treatment promoted increased IFN-γ secretion, consistent with its ability to enhance NK cell activation and cytotoxicity in vitro. These findings suggest that bacRNA, acting as a TLR8 agonist, not only enhances the NK cytotoxic response but also acts as an adjuvant. Taken together, these effects of bacRNA could contribute to the observed delay in tumor growth.” (Page 24, lines 561-568 of the revised version of the manuscript).

43. Zhou J, Xu Y, Wang G, Mei T, Yang H, Liu Y. The TLR7/8 agonist R848 optimizes host and tumor immunity to improve therapeutic efficacy in murine lung cancer. Int J Oncol. 2022;61. doi:10.3892/IJO.2022.5371

46. Kim H, Khanna V, Kucaba TA, Zhang W, Sehgal D, Ferguson DM, et al. TLR7/8 Agonist-Loaded Nanoparticles Augment NK Cell-Mediated Antibody-Based Cancer Immunotherapy. Mol Pharm. 2020;17: 2109–2124. doi:10.1021/ACS.MOLPHARMACEUT.0C00271

47. Rodell CB, Arlauckas SP, Cuccarese MF, Garris CS, Li R, Ahmed MS, et al. TLR7/8-agonist-loaded nanoparticles promote the polarization of tumour-associated macrophages to enhance cancer immunotherapy. Nat Biomed Eng. 2018;2: 578–588. Doi:10.1038/S41551-018-0236-8

**6.** Did authors verify MHC-1 downregulation in freshly isolated tumors injected with bRNA? This data is crucial for confirmation of in vitro results presented in Fig 1.

We agree with the AE that measuring MHC-I down-modulation in freshly isolated tumors would have been appropriate to confirm the results presented in Figure 1, However, we have no markers to stain the tumor cells specifically. We have tried to separate them with FSC *vs* SSC, but it was tedious and diffuse and we do not consider showing these results in the manuscript. As the AE may know, Argentina is facing an unprecedented scientific crisis and we (and so many other laboratories) hardly have the funds to purchase flow cytometry markers.

**Reviewer #1:** The manuscript title “Bacterial RNA downregulates MHC-I in tumor cell lines, promoting NK response and delaying tumor growth” by Serafino A et al., aimed to examine how bacterial RNA from Brucella abortus influences MHC-I expression in tumors, with the goal of enhancing NK cell activity and strengthening anti-tumor immune responses. To address this, the authors conducted cell culture experiments using multiple in vitro cancer cell lines to investigate how bacterial RNA downregulates IFN-γ–induced MHC-I expression in tumor cells, followed by in vivo studies. Overall, the manuscript is well written, and my comments are given below:

**Comments:**

Line 65-67: Here, the authors have addressed the role of NK cells rather superficially, relying on a general review in immuno-oncology that is not directly aligned with the central theme of the manuscript—namely, how enhanced NK cell activity promotes anti-tumor responses and the contribution of pathogenic material to this effect. It would therefore strengthen the manuscript to include a more detailed introduction on the importance of NK cells, their specific roles across different cancer types, and how pathogenic factors influence these responses, supported by relevant bacterial or pathogen examples.

We agree with the reviewer that a thorough introduction on the importance of how bacteria influence NK responses would strengthen the manuscript. We added the following paragraphs to the introduction [Page 3 (lines 66-68) and page 4 (lines 69-86) of the revised version of the manuscript]:

Natural killer (NK) cells are potent cytotoxic innate immune cells that play a fundamental role in tumor immunosurveillance by directly eliminating transformed cells and producing proinflammatory cytokines. However, in the immunosuppressive tumor microenvironment, NK cells become dysfunctional through exposure to inhibitory molecules produced by cancer cells, leading to tumor escape [5,6].

NK perform diverse roles across malignancies; for instance, robust NK cell infiltration is associated with prolonged survival in colorectal carcinoma, melanoma, and early-stage non-small cell lung cancer (NSCLC), whereas their dysfunction or decreased numbers are linked to disease progression in gastric, prostate, hepatocellular, and breast cancers. These anti-tumor responses are significantly influenced by pathogenic factors, particularly through the stimulation of Toll-like receptors (TLRs) by microbial-derived ligands [5,7].

For instance, the TLR5 agonist entolimod (former name CBLB502) rapidly activates TLR5-NF-kB signaling in hepatocytes and suppresses growth of both TLR5-expressing and non-expressing tumors in the liver through mobilization and activation of innate and adaptive immune mechanisms [8]. Similarly, priming with Bacillus Calmette-Guérin (BCG) can rescue dysfunctional NK cells from cancer patients, expanding a unique, long-lived CD56highCD16^+^ population that exerts potent cytotoxic activity against various solid tumors through NKG2D activation [6]. Furthermore, viral-derived factors, such as adenoviral CpG islands, can enhance anti-tumor immunity by stimulating TLR9 to increase NK cell-mediated killing of tumor cells [7]. These examples demonstrate how pathogen-associated molecular patterns could effectively modulate NK cell responses to overcome the immunosuppressive tumor microenvironment.

5. Cózar, B., Greppi, M., Carpentier, S., Narni-Mancinelli, E., Chiossone, L., & Vivier, E. (2021). Tumor-Infiltrating Natural Killer Cells. *Cancer discovery*, *11*(1), 34–44. [https://doi.org/10.1158/2159-8290.CD-20-0655](https://doi.org/10.1158/2159-8290.CD-20-0655%20%0d2)

6. Felgueres, M. J., Esteso, G., García-Jiménez, Á. F., Dopazo, A., Aguiló, N., Mestre-Durán, C., Martínez-Piñeiro, L., Pérez-Martínez, A., Reyburn, H. T., & Valés-Gómez, M. (2024). BCG priming followed by a novel interleukin combination activates Natural Killer cells to selectively proliferate and become anti-tumour long-lived effectors. *Scientific reports*, *14*(1), 13133. [https://doi.org/10.1038/s41598-024-62968-2](https://doi.org/10.1038/s41598-024-62968-2%20%0d3)

[7.](https://doi.org/10.1038/s41598-024-62968-2%20%0d3) Giurini, E. F., Madonna, M. B., Zloza, A., & Gupta, K. H. (2022). Microbial-Derived Toll-like Receptor Agonism in Cancer Treatment and Progression. *Cancers*, *14*(12), 2923. [https://doi.org/10.3390/cancers14122923](https://doi.org/10.3390/cancers14122923%20%0d4)

8. Yang, H., Brackett, C. M., Morales-Tirado, V. M., Li, Z., Zhang, Q., Wilson, M. W., Benjamin, C., Harris, W., Waller, E. K., Gudkov, A. V., Burdelya, L. G., & Grossniklaus, H. E. (2016). The Toll-like receptor 5 agonist entolimod suppresses hepatic metastases in a murine model of ocular melanoma via an NK cell-dependent mechanism. Oncotarget, 7(3), 2936–2950. <https://doi.org/10.18632/oncotarget.6500>

Line 82: Here, it would be helpful to specify which other bacterial RNAs can elicit similar effects, providing appropriate examples to support this phenomenon.

We apologize for omitting this information, as it is included in Supplementary Figure 6 of our previously published article (Milillo MA et al. *PLoS Pathogens,* 13(8): e1006527, 2017. doi: 10.1371/journal.ppat.1006527. <https://doi.org/10.1371/journal.ppat.1006527>). In this article, we demonstrated that the RNA of *Salmonella typhimurium, Bacillus cereus*, *Escherichia coli* and *Klebsiella pneumonia* but not eukaryotic RNAs (derived from PBMCs) are also able to inhibit IFN-γ-induced MHC-I expression (<https://doi.org/10.1371/journal.ppat.1006527.s006>). So, as suggested by the reviewer, we included the RNAs of these bacteria as examples of other bacterial RNAs capable of inhibiting MHC-I in the revised version of the manuscript (Page 5, lines 104-105).

Line 118: Here, bacterial strains other than Brucella are mentioned, but they are neither used nor further discussed in the manuscript. Please clarify the rationale for including these strains.

As mentioned in the previous paragraph and included in the manuscript, the RNAs from both *Escherichia coli* and *Klebsiella pneumoniae* mimic the down-regulation of IFN-γ-induced MHC-I surface expression mediated by *Brucella abortus* RNA. For this reason, in this study we hypothesize that bacterial RNA (bacRNA), and not only the RNA from *B. abortus*, can be used to modulate MHC-I expression in tumors to enhance the NK cell response. To test our hypothesis, as indicated in the Materials and Methods section (“Bacterial strains”), we performed bacterial cultures of *B. abortus,* *Escherichia coli* and *Klebsiella pneumoniae*. Additionally, RNA was purified from each of the three bacterial strains and subsequently pooled; these RNA preparations were generically named “bacRNA.” This pooled bacRNA preparation was used throughout the study. To clarify this point, we have included the corresponding description in the Materials and Methods section (“RNA Preparation”) of the revised version of the manuscript (Page 8, lines 171-172 and lines 176-178).

Line 150: Clarify whether this synthetic hTLR8 agonist was purchased commercially; if so, please include the company name. Also, IFN-γ concentration is not consistently indicated throughout the manuscript. Please specify the concentration used in the experiments, whether multiple concentrations were tested, and if so, whether a dose-dependent effect was observed with increasing IFN-γ levels.

The synthetic hTLR8 agonist was purchased commercially from *InvivoGen*. The product specifications are as follows:

ORN06/LyoVec™ - TLR7/8 Agonist (UUGU ssRNA polymer / LyoVec™ complexed). Cat.code: tlrl-orn6. <https://www.invivogen.com/orn06-lv#block_documents>

As suggested by the reviewer, the name of the company in which we purchased the synthetic hTLR8 agonist was included in the revised version of the manuscript (Page 8, line 182).

The final concentration of IFN-γ used throughout all experiments in the manuscript was 7.5 ng/ml. As suggested by the reviewer, this concentration was included the first time the use of IFN-γ was mentioned in the Materials and Methods section (“Viability assay”) in the revised version of the manuscript (Page 8, line 183).

Line 291 – 299 (Fig 4): Here, the authors state that PBMCs from healthy donors were used as effector cells but later indicate that NK cells were defined and used at a 3:1 ratio in the co-culture experiments. It is unclear whether the co-cultures were performed with total PBMCs or with purified NK cells. If NK cells were used, please clarify the method employed for their isolation or sorting, and how many cells were used for each donor and how did they normalize for each donor experiments?

To clarify this point, the cytotoxicity assays shown in the original version of the manuscript were performed using whole PBMCs. For these assays, PBMC normalized by the percentage of NK cells were used as effector cells. The percentage of NK cells was determined before the experiment for each donor, so that equivalent effector NK cell-to-target ratios were set up for the different donors. However, to strength the cytotoxicity results obtained using whole PBMCs (corresponding to Figure 4 in the original version of the manuscript) and as suggested by the AE and the reviewer 2, we have performed cytotoxicity experiments with purified NK cells using the U251 tumor line. NK cells were isolated from PBMC using the NK cell Isolation Kit (Miltenyi Biotec). Our cytotoxicity experiments with purified NK cells replicated the results we had reported in the original version of the manuscript using whole PBMCs. These results confirm, at least for the U251 tumor line, that treatment with bacRNA effectively enhances the cytotoxicity of NK cells. These results have been incorporated as Panel D in Figure 3 of the revised version of the manuscript. Furthermore, as suggested by the reviewer 2, we performed NK cell activation experiments using the U251 tumor line. In these assays, we evaluated both CD107a degranulation and IFN-γ production. Our results showed that not only does treatment with bacRNA increase the cytotoxic capacity of NK cells, but also activate them, as evidenced by greater CD107a degranulation and an increased IFN-γ production. These results have been incorporated as panels E and F in Figure 3 of the revised version of the manuscript. We have added a paragraph describing these results in the Results section (Page 16, lines 361–370 of the revised version of the manuscript). Additionally, we expanded the “Cell cultures” paragraph [Page 7 (lines 163-165) and page 8 (line 166) of the revised version of the manuscript], the “Cytotoxicity assays” paragraph (Page 10, lines 221-230 of the revised version of the manuscript) and wrote a new “NK activation assay” paragraph in the Materials and Methods section [Page 10 (lines 232–236) and page 11 (237-245) of the revised version of the manuscript]. Moreover, we have extended the legend of Figure 3 (Page 17, lines 379-385 of the revised version of the manuscript).

The cytotoxicity experiments with purified NK cells were conducted in collaboration with María Belén Bordignon and Ayelén Ivana Pesce Viglietti from “Centro de Investigaciones Oncológicas-Fundación Cáncer FUCA (Buenos Aires, Argentina)”. For this reason, they were added as authors to the revised version of the manuscript.

Additionally, in Figure 4, if the co-culture was initiated with NK cells at a 3:1 effector-to-target ratio, it is not clear whether the reported % CFSE proliferation corresponds to NK cells or tumor cells. Please clarify how these populations were distinguished and how enhanced NK cell cytotoxicity was determined.

We understand that the cytotoxicity assay may not have been entirely clear. To clarify the reviewer’s comment, the reported % Zombie Violet^+^ of CFSE^+^ cells correspond to tumor cells. In cytotoxicity assays, tumor cells (*i.e.*, the target cells) are initially labeled with CFSE. These CFSE-labeled target cells are then co-incubated with PBMCs (effector cells) for 5 hours. The CFSE labeling is what allows us to distinguish tumor cells from effector cells. After 5 hours of co-culture, the cells are stained with the cell viability marker Zombie Violet. The percentage of cytotoxicity is calculated as the % of Zombie Violet-positive cells relative to the total number of CFSE-positive cells. In other words, this percentage indicates the percentage of tumor cells (CFSE-positive cells) that are dead (Zombie Violet-positive cells). To clarify this point, we included a sentence in the “Cytotoxicity assays” paragraph in the Materials and Methods section (Page 10, lines 217-218 of the revised version of the manuscript).

Line 320: Here, the authors state that BacRNA promotes the infiltration of immune cells into the tumor microenvironment….. It would be important to clarify whether these cells are actively recruited. If so, did the authors use specific markers to characterize these populations—for example, inflammatory monocyte markers such as Ly6C, chemokine receptors like CCR2, or non-classical (patrolling) monocyte markers such as CX3CR1—to confirm and define the infiltrating immune cells? Additionally, the authors may wish to comment on whether such immune cell recruitment could lead to excessive inflammation, potentially contributing to pathogenesis and causing adverse effects.

We agree with the reviewer in the importance of measuring the activation of the infiltrating cells within the tumor. To evaluate this, we repeated the *in vivo* experiments with bacRNA (Figure 5 of the original version of the manuscript) but also included markers for the activation status of the cells. The activation markers used were as follows: - For DCs (CD11c^+^): MHC-II; - For monocytes (CD11b^+^): CXCR4; - For NK cells (NK1.1^+^): NKG2D; - For Th cells (CD4^+^): CD25; and - For CD8 T cells (CD8^+^): CD25. Our results showed that in mice treated with bacRNA, there is a significant activation in NK and Th cells. We also observed a tendency toward activation of DCs, monocytes, and CD8 T cells. These results were incorporated into panels C (for DCs), D (for monocytes), E (for NK cells), F (for Th cells), and G (for CD8 T cells) in Figure 4 of the revised version of the manuscript. We have also added a paragraph describing these results in the Results section (Page 18, lines 401–407 of the revised version of the manuscript). Additionally, we included information on the new antibodies in the Materials and Methods section (“Identification of immune cells in solid tumors”) in the revised version of the manuscript (Page 12, lines 275–283). Moreover, we have expanded the legend of Figure 4 (Page 18, lines 421-423 of the revised version of the manuscript).

On the other hand, due to the large number of panels included in Figure 4, the results showing the tumor size at different time points post-treatment with bacRNA have been moved to a separate figure (Figure 5 of the revised version of the manuscript) and therefore also have a separate figure legend (Page 19, lines 428-433 of the revised version of the manuscript).

Line 329 – 331: This statement appears overstated, as the study does not directly establish a mechanistic link between increased IFN-γ secretion and delayed tumor growth, yet it is presented as such here.

We thank the reviewer for this insightful comment and agree that our original statement may have overstated the relationship between IFN-γ secretion and tumor growth delay. We have revised the text to avoid implying a direct mechanistic link. While our data show that bacRNA treatment is associated with increased immune cell infiltration and elevated IFN-γ levels in the tumor microenvironment, we do not claim that IFN-γ is solely responsible for the observed reduction in tumor growth. As suggested by the reviewer, we have reformulated the sentence so that the revised version of the manuscript (Page 18, lines 414-416) now more accurately reflects a possible association rather than a causality.

Fig 5D: It is not entirely clear how the ELISA was performed in this murine melanoma (skin cancer) model. At the experimental endpoint, tumors were excised for downstream analyses; therefore, it is unclear how the authors obtained supernatants for the IFN-γ ELISA. Please clarify the source of the samples used for this assay. Also, the rationale for focusing solely on IFN-γ is not provided. It would strengthen the study to justify this choice or to include analysis of other inflammatory cytokines for a more comprehensive assessment of the immune response.

We apologize for not providing a clearer description of the procedure. To address the reviewer’s comment, after obtaining single-cell suspensions from solid melanoma tumors, 500,000 cells were seeded in 96-well plates and incubated overnight. The cells were then collected and transferred to microcentrifuge tubes, centrifuged for 20 seconds, and the supernatants were carefully harvested. Finally, IFN-γ levels were determined in the tumor cell supernatants by ELISA, according to the manufacturer’s instructions (BD OptEIA Mouse IFN-γ ELISA Kit, Cat. No. 555138). To clarify this point, we included this information in the section of Material and Methods “Measurement of extracellular IFN-γ” of the revised version of the manuscript (Page 13, lines 288-293).

In response to the reviewer’s concern regarding the rationale for focusing on IFN-γ, we selected this cytokine because it is a hallmark of NK cell activation and a key mediator of Th1-type immune responses. Therefore, its measurement may provide an indication of NK cell activity and the induction of a pro-inflammatory immune response, potentially contributing to the recruitment and infiltration of other immune cells into the tumor microenvironment.

Fig 5: Did the authors perform similar in vivo experiments using synthetic hTLR8 agonists, given that BacRNA showed comparable effects? If so, please clarify whether these agonists recapitulate TLR8-mediated responses within the in vivo tumor microenvironment?

We agree with the reviewer on the importance of evaluating the effect of the hTLR8 ligand ORN06 *in vivo*, given the comparable effects of bacRNA and ORN06 *in vitro*.

In our manuscript, we had evaluated the effect of ORN06 on MHC-I expression in the U251, LN-229, and HT-29 cell lines, but not in the B16-OVA cell line. Therefore, we first evaluated the effect *in vitro* of the ORN06 on the B16-OVA cell line. We evaluated both the effect of ORN06 on MHC-I expression and on cell viability. Our results showed that, as with the other tumor cell lines evaluated, ORN06 downregulated IFN-γ-induced MHC-I surface expression in the B16-OVA tumor cell line without affecting its viability. These results have been incorporated as panel D in Figure 2 of the revised version of the manuscript. For its part, the viability results have been incorporated as Panel D in Supplementary Figure 2 of the revised version of the manuscript. Having confirmed the *in vitro* effect of ORN06, we then evaluated its *in vivo* effect. For this, we performed the same *in vivo* experiments on tumor progression but injected the ORN06 agonist instead of bacRNA. Our results showed that ORN06 tended to increase the percentage and activation of DCs. In turn, ORN06 tended to increase the percentage of monocytes, which exhibited increased expression of CXCR4. ORN06 increased the percentage of NK cells (NK1.1^+^ cells) and the expression of NKGD2. Moreover, ORN06 increased the percentage of Th cells and the expression of CD25. We also observed that ORN06 increased the percentage of CD8 T cells and tended to increase its activation marker. In addition, ORN06 tended to induce the secretion of IFN-γ as measured by ELISA in supernatants harvested from the tumor microenvironment. Finally, we observed that, at the endpoint, the tumor sizes of ORN06-treated mice were smaller than those of the control mice, although this difference was not statistically significant. Overall, these results indicate that ORN06 mimics the effects of bacterial RNA within the *in vivo* tumor microenvironment in the B16 melanoma model, but to a lesser extent than bacRNA. All of these new results have been added as Figure 6 of the revised version of the manuscript, for which a new figure legend has also been written (Page 20, lines 461-472 of the revised version of the manuscript). We have also added a paragraph describing these results in the Results section [Page 19 (lines 435–447) and page 20 (448-459) of the revised version of the manuscript].

**General comment:**

All figures appear blurry and lack sufficient resolution; therefore, it is recommended to replace them with higher-quality images.

As suggested by the reviewer we improved the figures in terms of image quality.

**Reviewer #2:** In the opinion of this reviewer, the manuscript titled 'Bacterial RNA downregulates MHC-I in tumor cell lines, promoting NK response and delaying tumor growth' by Serafino et al. is an interesting, sufficiently original, and well-written report that, once the 'Major Concerns' have been addressed by the authors, could be recommended for publication in PLOS ONE.

**Major Concerns**

Throughout the entire manuscript (e.g., Title; Intro, lines 95-96; M&M, lines 175-185; Results, lines 286-299; and Conclusions, lines 385-387), the authors claim that the “decrease in MHC-I mediated by bacRNA correlated with increased NK cytotoxicity.” This is a critical point regarding the translational implications of the work, as the authors state: “the potential for utilization of bacterial RNA (bacRNA) in the modulation of MHC-I within tumors, where the NK cell response is of particular significance” (lines 90-91).

The major concern of this reviewer is that the experiment used to support this claim (Figure 4) is not a strictly controlled NK-cytotoxicity assay. The role of NK cells in inducing the increased mortality observed after 5h of co-incubation with heterologous PBMCs should be clearly established, ruling out other possible causes (e.g., CD8+ T-cell mismatch, activated monocytes, -or even PMN contamination- activated by remaining traces IFN-γ used in pre-cocultures, etc.).

In the opinion of this reviewer, the authors should adopt a classical approach using purified NK cells in an effector-to-target (E:T) ratio gradient, instead of the fixed E:T=3 assay based on whole PBMCs. Another (better, though not ideal) option would be to demonstrate that the depletion of CD3–/CD56+ or NKp46+ cells significantly restores cell viability.

Moreover, the authors do not provide data regarding the activation state of the NK cells (e.g., degranulation via CD107a expression, or HLA-DR/CD69 up-regulation) after the 5h co-culture with tumor cell lines stimulated with bacRNA and IFN-γ.

Therefore, this reviewer considers it necessary to strengthen the results in Figure 4 with the suggested complementary experiments; otherwise, the authors should significantly moderate the discussion regarding NK cell-mediated cytotoxic activity.

Since our manuscript focuses on the cytotoxic activity of NK cells, we agree with the reviewer on the importance of strengthening the cytotoxicity results obtained using whole PBMCs (corresponding to Figure 4 in the original version of the manuscript). For this reason, we have performed cytotoxicity experiments with purified NK cells using the U251 tumor line. NK cells were isolated from PBMC using the NK cell Isolation Kit (Miltenyi Biotec). Our cytotoxicity experiments with purified NK cells replicated the results we had reported in the original version of the manuscript using whole PBMCs. These results confirm, at least for the U251 tumor line, that treatment with bacRNA effectively enhances the cytotoxicity of NK cells. These results have been incorporated as Panel D in Figure 3 of the revised version of the manuscript. Furthermore, as suggested by the reviewer, we performed NK cell activation experiments using the U251 tumor line. In these assays, we evaluated both CD107a degranulation and IFN-γ production. Our results showed that not only does treatment with bacRNA increase the cytotoxic capacity of NK cells, but also activate them, as evidenced by greater CD107a degranulation and an increased IFN-γ production. These results have been incorporated as panels E and F in Figure 3 of the revised version of the manuscript. We have added a paragraph describing these results in the Results section (Page 16, lines 361–370 of the revised version of the manuscript). Additionally, we expanded the “Cell cultures” paragraph [Page 7 (lines 163-165) and page 8 (line 166) of the revised version of the manuscript], the “Cytotoxicity assays” paragraph (Page 10, lines 221-230 of the revised version of the manuscript) and wrote a new “NK activation assay” paragraph in the Materials and Methods section [Page 10 (lines 232–236) and page 11 (237-245) of the revised version of the manuscript]. Moreover, we have extended the legend of Figure 3 (Page 17, lines 379-385 of the revised version of the manuscript).

The cytotoxicity experiments with purified NK cells were conducted in collaboration with María Belén Bordignon and Ayelén Ivana Pesce Viglietti from “Centro de Investigaciones Oncológicas-Fundación Cáncer FUCA (Buenos Aires, Argentina)”. For this reason, they were added as authors to the revised version of the manuscript.
