# Supplementary material for: Bacterial RNA downregulates MHC-I in tumor cell lines, promoting NK response and delaying tumor growth
Source: PLoS One. 2026 Sep 15;21(9):e0357999. doi: 10.1371/journal.pone.0357999 (PMC13577469; doi:10.1371/journal.pone.0357999)
Supplement: Supplementary file 2 [file pone.0357999.s005.docx]

**Response to Reviewer 2**

**Minor comment**

Figure 3D: I think that the Y-axis label contradicts the figure legend (line 383). The axis reads '% of Zombie Violet of CD56+ cells', whereas the legend defines the dead U251 cells as '% of Zombie Violet (+) of CD56(-) cells'. If it may be the case, please correct the axis label to accurately reflect the target population.

Additionally, U251 glioblastoma cells are of neuroectodermal origin and strongly express NCAM (CD56). This baseline expression can cause severe background staining or unexpected cross-reactivity depending on the CD56 antibody clone used to rule out effector cells (purified NKs). This directly impacts the accuracy of the target cell definition. Please provide an explanatory note in the text of M&M to clarify how this was controlled, thereby improving the reproducibility of the assay.

We apologize for the error in the Y-axis label of Figure 3D. The correct information is the one provided both in the figure legend and the paragraph titled “Cytotoxicity assays” in the Materials and Methods section. Both paragraphs explain that NK cytotoxicity was measured as the percentage of Zombie Violet-positive cells relative to the total number of CD56-negative cells. Therefore, as suggested by the reviewer, the Y-axis label in Figure 3D has been corrected to accurately reflect the target population.

Regarding the other point mentioned by the reviewer, we are aware that CD56 or NCAM (Neural Cell Adhesion Molecule) is a molecule that is not only strongly expressed in NK cells but it is also expressed in cells of neuroectodermal origin. For this reason, since U251 glioblastoma cells are larger and more granular than NK cells, our gating strategy was to first gate on tumor cells in an FSC *vs*. SSC dot plot. From this gate, we performed another dot plot of SSC *vs*. CD56 and selected the CD56-negative U251 cells from it. Finally, we determined the percentage of Zombie Violet-positive cells relative to the total number of CD56-negative U251 cells. To clarify this point, we included our gating strategy in the “Cytotoxicity assays” paragraph in the Materials and Methods section (Page 10, lines 226-229 of the revised version of the manuscript).
