# Supplementary material for: Bacterial RNA downregulates MHC-I in tumor cell lines, promoting NK response and delaying tumor growth
Source: PLoS One. 2026 Sep 15;21(9):e0357999. doi: 10.1371/journal.pone.0357999 (PMC13577469; doi:10.1371/journal.pone.0357999)
Supplement: Peer review history

# Peer review: Decision Letter 0

**DOI:** 10.1371/journal.pone.0357999.r001

**Authors:** Sriram Chitta

19 Apr 2026

Dear Dr. Barrionuevo,

Comments from both AE and Reviewers are given below.

Please submit your revised manuscript by Jun 03 2026 11:59PM. If you will need more time than this to complete your revisions, please reply to this message or contact the journal office at plosone@plos.org. When you're ready to submit your revision, log on to https://www.editorialmanager.com/pone/ and select the 'Submissions Needing Revision' folder to locate your manuscript file.

- A letter that responds to each point raised by the academic editor and reviewer(s). You should upload this letter as a separate file labeled 'Response to Reviewers'.
- A marked-up copy of your manuscript that highlights changes made to the original version. You should upload this as a separate file labeled 'Revised Manuscript with Track Changes'.
- An unmarked version of your revised paper without tracked changes. You should upload this as a separate file labeled 'Manuscript'.

If you would like to make changes to your financial disclosure, please include your updated statement in your cover letter. Guidelines for resubmitting your figure files are available below the reviewer comments at the end of this letter.

If applicable, we recommend that you deposit your laboratory protocols in protocols.io to enhance the reproducibility of your results. Protocols.io assigns your protocol its own identifier (DOI) so that it can be cited independently in the future. For instructions see: https://journals.plos.org/plosone/s/submission-guidelines#loc-laboratory-protocols. Additionally, PLOS ONE offers an option for publishing peer-reviewed Lab Protocol articles, which describe protocols hosted on protocols.io. Read more information on sharing protocols at https://plos.org/protocols?utm_medium=editorial-email&utm_source=authorletters&utm_campaign=protocols.

As the corresponding author, your ORCID iD is verified in the submission system and will appear in the published article. PLOS supports the use of ORCID, and we encourage all coauthors to register for an ORCID iD and use it as well. Please encourage your coauthors to verify their ORCID iD within the submission system before final acceptance, as unverified ORCID iDs will not appear in the published article. *Only*  the individual author can complete the verification step; PLOS staff *cannot*  verify ORCID iDs on behalf of authors.

We look forward to receiving your revised manuscript.

Kind regards,

Sriram Chitta, Ph D

Academic Editor

PLOS One

Journal Requirements:

When submitting your revision, we need you to address these additional requirements.

1. Please ensure that your manuscript meets PLOS ONE's style requirements, including those for file naming. The PLOS ONE style templates can be found at

https://journals.plos.org/plosone/s/file?id=wjVg/PLOSOne_formatting_sample_main_body.pdf and

https://journals.plos.org/plosone/s/file?id=ba62/PLOSOne_formatting_sample_title_authors_affiliations.pdf

2. We noticed you have some minor occurrence of overlapping text with the following previous publication(s), which needs to be addressed:

https://journals.lww.com/anti-cancerdrugs/abstract/2024/08000/targeting_hsp47_for_cancer_treatment.4.aspx

In your revision ensure you cite all your sources (including your own works), and quote or rephrase any duplicated text outside the methods section. Further consideration is dependent on these concerns being addressed.

3. Thank you for stating the following financial disclosure:

“This work was financed by PB PICT-2020 SERIE A-00978 and MAM PICT-2020 SERIE A-00882 grants from the Agencia Nacional de Promoción Científica y Tecnológica (ANPCYT-Argentina) and PB PIP 2021-2023. Nº 1220200100370CO from the Consejo Nacional de Investigaciones Científicas y Tecnológicas (CONICET-Argentina). At present, research funding in our country has been discontinued. As a result, the study presented here was made possible thanks to the resources obtained more than two years ago.”

Please state what role the funders took in the study.  If the funders had no role, please state: "The funders had no role in study design, data collection and analysis, decision to publish, or preparation of the manuscript."

If this statement is not correct you must amend it as needed.

Please include this amended Role of Funder statement in your cover letter; we will change the online submission form on your behalf.

4. Thank you for stating the following financial disclosure:

“This work was financed by PB PICT-2020 SERIE A-00978 and MAM PICT-2020 SERIE A-00882 grants from the Agencia Nacional de Promoción Científica y Tecnológica (ANPCYT-Argentina) and PB PIP 2021-2023. Nº 1220200100370CO from the Consejo Nacional de Investigaciones Científicas y Tecnológicas (CONICET-Argentina). At present, research funding in our country has been discontinued. As a result, the study presented here was made possible thanks to the resources obtained more than two years ago.”

We note that one or more of the authors is affiliated with the funding organization, indicating the funder may have had some role in the design, data collection, analysis or preparation of your manuscript for publication; in other words, the funder played an indirect role through the participation of the co-authors. If the funding organization did not play a role in the study design, data collection and analysis, decision to publish, or preparation of the manuscript and only provided financial support in the form of authors' salaries and/or research materials, please do the following:

1. Review your statements relating to the author contributions, and ensure you have specifically and accurately indicated the role(s) that these authors had in your study. These amendments should be made in the online form.

2. Confirm in your cover letter that you agree with the following statement, and we will change the online submission form on your behalf:

“The funder provided support in the form of salaries for authors [insert relevant initials], but did not have any additional role in the study design, data collection and analysis, decision to publish, or preparation of the manuscript. The specific roles of these authors are articulated in the ‘author contributions’ section.

5. Thank you for uploading your study's underlying data set. Unfortunately, the repository you have noted in your Data Availability statement does not qualify as an acceptable data repository according to PLOS's standards.

At this time, please upload the minimal data set necessary to replicate your study's findings to a stable, public repository (such as figshare or Dryad) and provide us with the relevant URLs, DOIs, or accession numbers that may be used to access these data. For a list of recommended repositories and additional information on PLOS standards for data deposition, please see https://journals.plos.org/plosone/s/recommended-repositories.

6. When completing the data availability statement of the submission form, you indicated that you will make your data available on acceptance. We strongly recommend all authors decide on a data sharing plan before acceptance, as the process can be lengthy and hold up publication timelines. Please note that, though access restrictions are acceptable now, your entire data will need to be made freely accessible if your manuscript is accepted for publication. This policy applies to all data except where public deposition would breach compliance with the protocol approved by your research ethics board. If you are unable to adhere to our open data policy, please kindly revise your statement to explain your reasoning and we will seek the editor's input on an exemption. Please be assured that, once you have provided your new statement, the assessment of your exemption will not hold up the peer review process.

7. If the reviewer comments include a recommendation to cite specific previously published works, please review and evaluate these publications to determine whether they are relevant and should be cited. There is no requirement to cite these works unless the editor has indicated otherwise.

Additional Editor Comments:

Manuscript entitled " Bacterial RNA downregulation MHC-I in tumor cell lines, promoting NK response and delaying tumor growth), submitted by Serafino et al is thoroughly reviewed by two experts related to the main topic of manuscript. Based on the reports submitted and personal analysis, I would like to state that the manuscript is not acceptable in current form and requires major revision, to get considered as PONE publication. I am including the comment from reviewers, along with my own, for the convenience of authors, in revising the manuscript. Incidentally, reviewers and AE suggestions may involve running couple of experiments to sharpen the findings presented/discussed and increase quality of paper.

Best Regards

Sriram Chitta

Comments:

AE:

1. Authors should show MHC I down regulation by bRNA in graded concentrations, by selecting one low (no inhibition) and one high (max inhibition). The data showed has concentrations in plateau region.

2. MFI/SEM values on Y axis of fig 1 & 3: Can you show a representative GM value for isotype, MHC-1 in presence and absence of bRNA. The value showed appears as "fold" change.

3. Viability figs can go as Supplement figs as they are not adding any additional information

4. (Same as reviewer 2): Since the main point of manuscript deals with NK cell activity, the experiment shown in manuscript is not In-depth one. When PBMC were used as effector cells, percentage of NK cells will be very low in numbers. it is advised to use PBMC in graded doses, to measure killing activity. Authors are advised to re-run this experiment, as suggested.

5. All immune cells (CD4, DC, Monocytes, CD8 T cells (authors wrongly used as CTL?) increased with bRNA injection in tumors. There is no specific increase in NK cells alone. Can authors comment on?

6. Did authors verify MHC-1 downregulation in freshly isolated tumors injected with bRNA? This data is crucial for confirmation of invitro results presented in Fig 1.

[Note: HTML markup is below. Please do not edit.]

Reviewers' comments:

Reviewer's Responses to Questions

**Comments to the Author**

1. Is the manuscript technically sound, and do the data support the conclusions?

Reviewer #1: Partly

Reviewer #2: Partly

**********

2. Has the statistical analysis been performed appropriately and rigorously?

Reviewer #1: Yes

Reviewer #2: Yes

**********

3. Have the authors made all data underlying the findings in their manuscript fully available?

The PLOS Data policy

Reviewer #1: Yes

Reviewer #2: Yes

**********

4. Is the manuscript presented in an intelligible fashion and written in standard English?

Reviewer #1: Yes

Reviewer #2: Yes

**********

Reviewer #1: The manuscript title “Bacterial RNA downregulates MHC-I in tumor cell lines, promoting NK response and delaying tumor growth” by Serafino A et al., aimed to examine how bacterial RNA from Brucella abortus influences MHC-I expression in tumors, with the goal of enhancing NK cell activity and strengthening anti-tumor immune responses. To address this, the authors conducted cell culture experiments using multiple in vitro cancer cell lines to investigate how bacterial RNA downregulates IFN-γ–induced MHC-I expression in tumor cells, followed by in vivo studies. Overall, the manuscript is well written, and my comments are given below:

Comments:

Line 65-67: Here, the authors have addressed the role of NK cells rather superficially, relying on a general review in immuno-oncology that is not directly aligned with the central theme of the manuscript—namely, how enhanced NK cell activity promotes anti-tumor responses and the contribution of pathogenic material to this effect. It would therefore strengthen the manuscript to include a more detailed introduction on the importance of NK cells, their specific roles across different cancer types, and how pathogenic factors influence these responses, supported by relevant bacterial or pathogen examples.

Line 82: Here, it would be helpful to specify which other bacterial RNAs can elicit similar effects, providing appropriate examples to support this phenomenon.

Line 118: Here, bacterial strains other than Brucella are mentioned, but they are neither used nor further discussed in the manuscript. Please clarify the rationale for including these strains.

Line 150: Clarify whether this synthetic hTLR8 agonist was purchased commercially; if so, please include the company name. Also, IFN-γ concentration is not consistently indicated throughout the manuscript. Please specify the concentration used in the experiments, whether multiple concentrations were tested, and if so, whether a dose-dependent effect was observed with increasing IFN-γ levels.

Line 291 – 299 (Fig 4): Here, the authors state that PBMCs from healthy donors were used as effector cells but later indicate that NK cells were defined and used at a 3:1 ratio in the co-culture experiments. It is unclear whether the co-cultures were performed with total PBMCs or with purified NK cells. If NK cells were used, please clarify the method employed for their isolation or sorting, and how many cells were used for each donor and how did they normalize for each donor experiments?

Additionally, in Figure 4, if the co-culture was initiated with NK cells at a 3:1 effector-to-target ratio, it is not clear whether the reported % CFSE proliferation corresponds to NK cells or tumor cells. Please clarify how these populations were distinguished and how enhanced NK cell cytotoxicity was determined.

Line 320: Here, the authors state that BacRNA promotes the infiltration of immune cells into the tumor microenvironment….. It would be important to clarify whether these cells are actively recruited. If so, did the authors use specific markers to characterize these populations—for example, inflammatory monocyte markers such as Ly6C, chemokine receptors like CCR2, or non-classical (patrolling) monocyte markers such as CX3CR1—to confirm and define the infiltrating immune cells? Additionally, the authors may wish to comment on whether such immune cell recruitment could lead to excessive inflammation, potentially contributing to pathogenesis and causing adverse effects.

Line 329 – 331: This statement appears overstated, as the study does not directly establish a mechanistic link between increased IFN-γ secretion and delayed tumor growth, yet it is presented as such here.

Fig 5D: It is not entirely clear how the ELISA was performed in this murine melanoma (skin cancer) model. At the experimental endpoint, tumors were excised for downstream analyses; therefore, it is unclear how the authors obtained supernatants for the IFN-γ ELISA. Please clarify the source of the samples used for this assay. Also, the rationale for focusing solely on IFN-γ is not provided. It would strengthen the study to justify this choice or to include analysis of other inflammatory cytokines for a more comprehensive assessment of the immune response.

Fig 5: Did the authors perform similar in vivo experiments using synthetic hTLR8 agonists, given that BacRNA showed comparable effects? If so, please clarify whether these agonists recapitulate TLR8-mediated responses within the in vivo tumor microenvironment?

General comment:

All figures appear blurry and lack sufficient resolution; therefore, it is recommended to replace them with higher-quality images.

Reviewer #2: In the opinion of this reviewer, the manuscript titled 'Bacterial RNA downregulates MHC-I in tumor cell lines, promoting NK response and delaying tumor growth' by Serafino et al. is an interesting, sufficiently original, and well-written report that, once the 'Major Concerns' have been addressed by the authors, could be recommended for publication in PLOS ONE.

Major Concerns

Throughout the entire manuscript (e.g., Title; Intro, lines 95-96; M&M, lines 175-185; Results, lines 286-299; and Conclusions, lines 385-387), the authors claim that the “decrease in MHC-I mediated by bacRNA correlated with increased NK cytotoxicity.” This is a critical point regarding the translational implications of the work, as the authors state: “the potential for utilization of bacterial RNA (bacRNA) in the modulation of MHC-I within tumors, where the NK cell response is of particular significance” (lines 90-91).

The major concern of this reviewer is that the experiment used to support this claim (Figure 4) is not a strictly controlled NK-cytotoxicity assay. The role of NK cells in inducing the increased mortality observed after 5h of co-incubation with heterologous PBMCs should be clearly established, ruling out other possible causes (e.g., CD8+ T-cell mismatch, activated monocytes, -or even PMN contamination- activated by remaining traces IFN-γ used in pre-cocultures, etc.).

In the opinion of this reviewer, the authors should adopt a classical approach using purified NK cells in an effector-to-target (E:T) ratio gradient, instead of the fixed E:T=3 assay based on whole PBMCs. Another (better, though not ideal) option would be to demonstrate that the depletion of CD3–/CD56+ or NKp46+ cells significantly restores cell viability.

Moreover, the authors do not provide data regarding the activation state of the NK cells (e.g., degranulation via CD107a expression, or HLA-DR/CD69 up-regulation) after the 5h co-culture with tumor cell lines stimulated with bacRNA and IFN-γ.

Therefore, this reviewer considers it necessary to strengthen the results in Figure 4 with the suggested complementary experiments; otherwise, the authors should significantly moderate the discussion regarding NK cell-mediated cytotoxic activity.

**********

what does this mean?). If published, this will include your full peer review and any attached files.

If you choose “no”, your identity will remain anonymous but your review may still be made public.

**Do you want your identity to be public for this peer review?**  For information about this choice, including consent withdrawal, please see our Privacy Policy

Reviewer #1: No

Reviewer #2: **Yes:** Pablo Schierloh

**********

[NOTE: If reviewer comments were submitted as an attachment file, they will be attached to this email and accessible via the submission site. Please log into your account, locate the manuscript record, and check for the action link "View Attachments". If this link does not appear, there are no attachment files.]

To ensure your figures meet our technical requirements, please review our figure guidelines: https://journals.plos.org/plosone/s/figures

You may also use PLOS’s free figure tool, NAAS, to help you prepare publication quality figures: https://journals.plos.org/plosone/s/figures#loc-tools-for-figure-preparation.

NAAS will assess whether your figures meet our technical requirements by comparing each figure against our figure specifications.

# Author comment: Author response to Decision Letter 1

**DOI:** 10.1371/journal.pone.0357999.r002

16 Jul 2026

Comments:

Academic Editor (AE):

1. Authors should show MHC I down regulation by bRNA in graded concentrations, by selecting one low (no inhibition) and one high (max inhibition). The data showed concentrations in plateau region.

As suggested by the AE, we now show MHC-I down-regulation by bacRNA in one low (1 μg/ml) and one high (10 μg/ml) concentration (Figure 1 of the revised version of the manuscript).

2. MFI/SEM values on Y axis of fig 1 & 3: Can you show a representative GM value for isotype, MHC-1 in presence and absence of bRNA. The value showed appears as "fold" change.

As mentioned by the AE, the data were presented as a “fold” change to minimize dispersion among the data obtained in the independent experiments. However, as suggested by the AE, we present the mean fluorescence intensity (MFI) values corresponding to all the graphs in Figure 1 and Figure 2 (which was Figure 3 in the original version of the manuscript). To organize these values, we designed the tables shown below. Given that raw data has been uploaded to the Zenodo public repository (https://doi.org/10.5281/zenodo.21107026), we decided not to add these tables onto the final version of the manuscript.

Figure 1 RAW DATA

- IFN-γ bacRNA 1 + IFN-γ bacRNA 10 + IFN-γ

U251 16.70 75.1 59 48.5

22.10 132 82.96 78.2

18.5 109.95 89.17 76.25

20.3 119.83 83.88 80.13

19.6 165.93 136.98 119.16

LN-229 58.4 256.51 176.99 208.22

62.2 204 194 182

76.4 186 176 173

33.9 124 90.3 95

17.2 44.2 44.8 37.6

40 99.1 112.74 92.6

55 218.9 197.62 179.25

HT-29 94.9 406 341 304

79.7 177 276 126

35.5 189 139 104

32.8 170 119 78.4

HCT 51 116 133 119

47 107 113 119

43.2 99.7 115 82.3

59.9 152 190 196

50.2 171 148 169

MCF-7 16.7 124 119 104

16.2 146 119 106

14.8 135 164.72 118.71

16.5 165 156.99 147.5

12.2 114 111 96.5

B16-OVA 13 122.4 117 92.4

19.86 130 125 118.6

16.4 101.99 86.99 73.5

17.9 107.52 104.53 85.12

Figure 2 RAW DATA

- IFN-γ bacRNA 10 + IFN-γ ORN06 + IFN-γ

U251 16.70 75.1 48.5

22.10 132 78.2

18.5 109.95 76.25

20.3 119.83 80.13 66.505

19.6 165.93 119.16 94.325

21.8 139.72 104.2 80.78

LN-229 76.4 186 173 141.378

58.4 256.51 208.22 161.4

62.2 204 182 147.15

33.9 124 95

55 218.9 179.25

HT-29 94.9 406 304 297.43

87.4 281.5 138.17 160.48

35.5 189 104 100.66

32.8 170 78.4

B16-OVA 13 122.4 92.4 55.79

19.86 130 118.6 59.99

16.4 101.99 73.5 82.1148

17.9 107.52 85.12

3. Viability figs can go as Supplement figs as they are not adding any additional information

As suggested by the AE, the cell viability figures (Figure 2 and Panels D, E and F of Figure 3 in the original version of the manuscript) have been moved to the Supporting information section (Figure S1 and Figure S2 of the revised version of the manuscript).

4. (Same as reviewer 2): Since the main point of manuscript deals with NK cell activity, the experiment shown in manuscript is not In-depth one. When PBMC were used as effector cells, percentage of NK cells will be very low in numbers. it is advised to use PBMC in graded doses, to measure killing activity. Authors are advised to re-run this experiment, as suggested.

Since our manuscript focuses on the cytotoxic activity of NK cells, we agree with the AE and the reviewer 2 on the importance of strengthening the cytotoxicity results obtained using whole PBMCs (corresponding to Figure 4 in the original version of the manuscript). For this reason, we have performed cytotoxicity experiments with purified NK cells using the U251 tumor line. NK cells were isolated from PBMC using the NK cell Isolation Kit (Miltenyi Biotec). Our cytotoxicity experiments with purified NK cells replicated the results we had reported in the original version of the manuscript using whole PBMCs. These results confirm, at least for the U251 tumor line, that treatment with bacRNA effectively enhances the cytotoxicity of NK cells. These results have been incorporated as Panel D in Figure 3 of the revised version of the manuscript. Furthermore, as suggested by the reviewer 2, we performed NK cell activation experiments using the U251 tumor line. In these assays, we evaluated both CD107a degranulation and IFN-γ production. Our results showed that not only does treatment with bacRNA increase the cytotoxic capacity of NK cells, but also activate them, as evidenced by greater CD107a degranulation and an increased IFN-γ production. These results have been incorporated as panels E and F in Figure 3 of the revised version of the manuscript. We have added a paragraph describing these results in the Results section (Page 16, lines 361–370 of the revised version of the manuscript). Additionally, we expanded the “Cell cultures” paragraph [Page 7 (lines 163-165) and page 8 (line 166) of the revised version of the manuscript], the “Cytotoxicity assays” paragraph (Page 10, lines 221-230 of the revised version of the manuscript) and wrote a new “NK activation assay” paragraph in the Materials and Methods section [Page 10 (lines 232–236) and page 11 (237-245) of the revised version of the manuscript]. Moreover, we have extended the legend of Figure 3 (Page 17, lines 379-385 of the revised version of the manuscript).

The cytotoxicity experiments with purified NK cells were conducted in collaboration with María Belén Bordignon and Ayelén Ivana Pesce Viglietti from “Centro de Investigaciones Oncológicas-Fundación Cáncer FUCA (Buenos Aires, Argentina)”. For this reason, they were added as authors to the revised version of the manuscript.

5. All immune cells (CD4, DC, Monocytes, CD8 T cells (authors wrongly used as CTL?) increased with bRNA injection in tumors. There is no specific increase in NK cells alone. Can authors comment on?

We agree with the AE that the increase not only in NK cells but also in other immune cells is a very important point that must be taken into account when interpreting and discussing the results. In fact, in the discussion section of the original manuscript, we dedicated a lengthy paragraph to the role of TLR 7/8 agonists as vaccine adjuvants in different tumor contexts [Page 23 (lines 527-545) and page 24 (lines 546-560) of the revised version of the manuscript]. Some of the cited studies mention: 1) Treatment with R848 resulted in increased levels of IFN-γ, TNF-α, and IL-2 as well as an increase in the proportion of DCs, NK, and CD8+ T cells, accompanied by a reduction in the proportion of Foxp3+ regulatory T cells within the tumor microenvironment [43] and 2) It has also been demonstrated that nanoparticles loaded with TLR7/8 agonists can be used as potent immunostimulatory adjuvants by promoting NK cell activation [46] and polarization of tumor-associated macrophages [47] to enhance cancer immunotherapy.

Furthermore, in our discussion, we explain the infiltration of various cell populations into the tumor microenvironment as follows:

“In line with the adjuvant capacity of TLR8 agonists, our in vivo experiments using a murine melanoma model showed that both bacRNA and the synthetic TLR8 agonist ORN06 promoted the infiltration and activation of multiple immune cell populations (DCs, monocytes, NK cells, Th cells, and CD8 T cells) into the tumor microenvironment. In addition, bacRNA treatment promoted increased IFN-γ secretion, consistent with its ability to enhance NK cell activation and cytotoxicity in vitro. These findings suggest that bacRNA, acting as a TLR8 agonist, not only enhances the NK cytotoxic response but also acts as an adjuvant. Taken together, these effects of bacRNA could contribute to the observed delay in tumor growth.” (Page 24, lines 561-568 of the revised version of the manuscript).

43. Zhou J, Xu Y, Wang G, Mei T, Yang H, Liu Y. The TLR7/8 agonist R848 optimizes host and tumor immunity to improve therapeutic efficacy in murine lung cancer. Int J Oncol. 2022;61. doi:10.3892/IJO.2022.5371

46. Kim H, Khanna V, Kucaba TA, Zhang W, Sehgal D, Ferguson DM, et al. TLR7/8 Agonist-Loaded Nanoparticles Augment NK Cell-Mediated Antibody-Based Cancer Immunotherapy. Mol Pharm. 2020;17: 2109–2124. doi:10.1021/ACS.MOLPHARMACEUT.0C00271

47. Rodell CB, Arlauckas SP, Cuccarese MF, Garris CS, Li R, Ahmed MS, et al. TLR7/8-agonist-loaded nanoparticles promote the polarization of tumour-associated macrophages to enhance cancer immunotherapy. Nat Biomed Eng. 2018;2: 578–588. Doi:10.1038/S41551-018-0236-8

6. Did authors verify MHC-1 downregulation in freshly isolated tumors injected with bRNA? This data is crucial for confirmation of in vitro results presented in Fig 1.

We agree with the AE that measuring MHC-I down-modulation in freshly isolated tumors would have been appropriate to confirm the results presented in Figure 1, However, we have no markers to stain the tumor cells specifically. We have tried to separate them with FSC vs SSC, but it was tedious and diffuse and we do not consider showing these results in the manuscript. As the AE may know, Argentina is facing an unprecedented scientific crisis and we (and so many other laboratories) hardly have the funds to purchase flow cytometry markers.

Reviewer #1: The manuscript title “Bacterial RNA downregulates MHC-I in tumor cell lines, promoting NK response and delaying tumor growth” by Serafino A et al., aimed to examine how bacterial RNA from Brucella abortus influences MHC-I expression in tumors, with the goal of enhancing NK cell activity and strengthening anti-tumor immune responses. To address this, the authors conducted cell culture experiments using multiple in vitro cancer cell lines to investigate how bacterial RNA downregulates IFN-γ–induced MHC-I expression in tumor cells, followed by in vivo studies. Overall, the manuscript is well written, and my comments are given below:

Comments:

Line 65-67: Here, the authors have addressed the role of NK cells rather superficially, relying on a general review in immuno-oncology that is not directly aligned with the central theme of the manuscript—namely, how enhanced NK cell activity promotes anti-tumor responses and the contribution of pathogenic material to this effect. It would therefore strengthen the manuscript to include a more detailed introduction on the importance of NK cells, their specific roles across different cancer types, and how pathogenic factors influence these responses, supported by relevant bacterial or pathogen examples.

We agree with the reviewer that a thorough introduction on the importance of how bacteria influence NK responses would strengthen the manuscript. We added the following paragraphs to the introduction [Page 3 (lines 66-68) and page 4 (lines 69-86) of the revised version of the manuscript]:

Natural killer (NK) cells are potent cytotoxic innate immune cells that play a fundamental role in tumor immunosurveillance by directly eliminating transformed cells and producing proinflammatory cytokines. However, in the immunosuppressive tumor microenvironment, NK cells become dysfunctional through exposure to inhibitory molecules produced by cancer cells, leading to tumor escape [5,6].

NK perform diverse roles across malignancies; for instance, robust NK cell infiltration is associated with prolonged survival in colorectal carcinoma, melanoma, and early-stage non-small cell lung cancer (NSCLC), whereas their dysfunction or decreased numbers are linked to disease progression in gastric, prostate, hepatocellular, and breast cancers. These anti-tumor responses are significantly influenced by pathogenic factors, particularly through the stimulation of Toll-like receptors (TLRs) by microbial-derived ligands [5,7].

For instance, the TLR5 agonist entolimod (former name CBLB502) rapidly activates TLR5-NF-kB signaling in hepatocytes and suppresses growth of both TLR5-expressing and non-expressing tumors in the liver through mobilization and activation of innate and adaptive immune mechanisms [8]. Similarly, priming with Bacillus Calmette-Guérin (BCG) can rescue dysfunctional NK cells from cancer patients, expanding a unique, long-lived CD56highCD16+ population that exerts potent cytotoxic activity against various solid tumors through NKG2D activation [6]. Furthermore, viral-derived factors, such as adenoviral CpG islands, can enhance anti-tumor immunity by stimulating TLR9 to increase NK cell-mediated killing of tumor cells [7]. These examples demonstrate how pathogen-associated molecular patterns could effectively modulate NK cell responses to overcome the immunosuppressive tumor microenvironment.

5. Cózar, B., Greppi, M., Carpentier, S., Narni-Mancinelli, E., Chiossone, L., & Vivier, E. (2021). Tumor-Infiltrating Natural Killer Cells. Cancer discovery, 11(1), 34–44. https://doi.org/10.1158/2159-8290.CD-20-0655

6. Felgueres, M. J., Esteso, G., García-Jiménez, Á. F., Dopazo, A., Aguiló, N., Mestre-Durán, C., Martínez-Piñeiro, L., Pérez-Martínez, A., Reyburn, H. T., & Valés-Gómez, M. (2024). BCG priming followed by a novel interleukin combination activates Natural Killer cells to selectively proliferate and become anti-tumour long-lived effectors. Scientific reports, 14(1), 13133. https://doi.org/10.1038/s41598-024-62968-2

7. Giurini, E. F., Madonna, M. B., Zloza, A., & Gupta, K. H. (2022). Microbial-Derived Toll-like Receptor Agonism in Cancer Treatment and Progression. Cancers, 14(12), 2923. https://doi.org/

Attachment Submitted filename: Response to Reviewers.docx

# Peer review: Decision Letter 1

**DOI:** 10.1371/journal.pone.0357999.r003

**Authors:** Sriram Chitta

13 Aug 2026

Dear Dr. Barrionuevo,

Thank you for submitting your manuscript to PLOS ONE. After careful consideration, we feel that it has merit but does not fully meet PLOS ONE’s publication criteria as it currently stands. Therefore, we invite you to submit a revised version of the manuscript that addresses the points raised during the review process.

Please submit your revised manuscript by Sep 27 2026 11:59PM. If you will need more time than this to complete your revisions, please reply to this message or contact the journal office at plosone@plos.org. When you're ready to submit your revision, log on to https://www.editorialmanager.com/pone/ and select the 'Submissions Needing Revision' folder to locate your manuscript file.

- A letter that responds to each point raised by the academic editor and reviewer(s). You should upload this letter as a separate file labeled 'Response to Reviewers'.
- A marked-up copy of your manuscript that highlights changes made to the original version. You should upload this as a separate file labeled 'Revised Manuscript with Track Changes'.
- An unmarked version of your revised paper without tracked changes. You should upload this as a separate file labeled 'Manuscript'.

If you would like to make changes to your financial disclosure, please include your updated statement in your cover letter. Guidelines for resubmitting your figure files are available below the reviewer comments at the end of this letter.

If applicable, we recommend that you deposit your laboratory protocols in protocols.io to enhance the reproducibility of your results. Protocols.io assigns your protocol its own identifier (DOI) so that it can be cited independently in the future. For instructions see: https://journals.plos.org/plosone/s/submission-guidelines#loc-laboratory-protocols. Additionally, PLOS ONE offers an option for publishing peer-reviewed Lab Protocol articles, which describe protocols hosted on protocols.io. Read more information on sharing protocols at https://plos.org/protocols?utm_medium=editorial-email&utm_source=authorletters&utm_campaign=protocols.

As the corresponding author, your ORCID iD is verified in the submission system and will appear in the published article. PLOS supports the use of ORCID, and we encourage all coauthors to register for an ORCID iD and use it as well. Please encourage your coauthors to verify their ORCID iD within the submission system before final acceptance, as unverified ORCID iDs will not appear in the published article. *Only*  the individual author can complete the verification step; PLOS staff *cannot*  verify ORCID iDs on behalf of authors.

We look forward to receiving your revised manuscript.

Kind regards,

Sriram Chitta

Academic Editor

PLOS One

Journal Requirements:

If the reviewer comments include a recommendation to cite specific previously published works, please review and evaluate these publications to determine whether they are relevant and should be cited. There is no requirement to cite these works unless the editor has indicated otherwise.

Please review your reference list to ensure that it is complete and correct. If you have cited papers that have been retracted, please include the rationale for doing so in the manuscript text, or remove these references and replace them with relevant current references. Any changes to the reference list should be mentioned in the rebuttal letter that accompanies your revised manuscript. If you need to cite a retracted article, indicate the article’s retracted status in the References list and also include a citation and full reference for the retraction notice.

Additional Editor Comments :

Hi Dr Paula Barrionuevo

Thank you for submitting the revised manuscript. The revised manuscript adequately answered reviewers' questions, with justified additional experimental results. Overall, the revisions have significantly improved the manuscript. Therefore, I recommend that the revised manuscript be provisionally accepted for publication, in PLOS ONE. One of the reviewers had a further genuine question (given below) to be clarified for final acceptance of the manuscript. Hope you soon attend this minor edit/ clarify the question and resubmit (no more review).

We look forward to having your findings published soon in PLOS ONE.

Best Regards

Sriram Chitta

AE

Minor comment (Reviewer 2)

Figure 3D: I think that the Y-axis label contradicts the figure legend (line 383). The axis reads '% of Zombie Violet of CD56+ cells', whereas the legend defines the dead U251 cells as '% of Zombie Violet (+) of CD56(-) cells'. If it may be the case, please correct the axis label to accurately reflect the target population.

Additionally, U251 glioblastoma cells are of neuroectodermal origin and strongly express NCAM (CD56). This baseline expression can cause severe background staining or unexpected cross-reactivity depending on the CD56 antibody clone used to rule out effector cells (purified NKs). This directly impacts the accuracy of the target cell definition. Please provide an explanatory note in the text of M&M to clarify how this was controlled, thereby improving the reproducibility of the assay.

[Note: HTML markup is below. Please do not edit.]

Reviewers' comments:

Reviewer's Responses to Questions

**Comments to the Author**

Reviewer #1: All comments have been addressed

Reviewer #2: (No Response)

**********

2. Is the manuscript technically sound, and do the data support the conclusions?

Reviewer #1: Yes

Reviewer #2: Yes

**********

3. Has the statistical analysis been performed appropriately and rigorously?

Reviewer #1: Yes

Reviewer #2: Yes

**********

4. Have the authors made all data underlying the findings in their manuscript fully available?

The PLOS Data policy

Reviewer #1: Yes

Reviewer #2: (No Response)

**********

5. Is the manuscript presented in an intelligible fashion and written in standard English?

Reviewer #1: Yes

Reviewer #2: (No Response)

**********

Reviewer #1: The authors have adequately addressed all of my queries and provided appropriate justifications. They have also conducted additional experiments and substantially revised the manuscript in response to the comments. Overall, the revisions have significantly improved the manuscript. Therefore, I recommend that the revised manuscript be accepted for publication. Thank you.

Reviewer #2: In this reviewer's opinion, the authors have adequately addressed the comments I raised in a previous round of review, and I therefore consider this manuscript acceptable for publication. However, one minor comment remains.

Minnor comment:

Figure 3D: I think that the Y-axis label contradicts the figure legend (line 383). The axis reads '% of Zombie Violet of CD56+ cells', whereas the legend defines the dead U251 cells as '% of Zombie Violet (+) of CD56(-) cells'. If it may be the case, please correct the axis label to accurately reflect the target population.

Additionally, U251 glioblastoma cells are of neuroectodermal origin and strongly express NCAM (CD56). This baseline expression can cause severe background staining or unexpected cross-reactivity depending on the CD56 antibody clone used to rule out effector cells (purified NKs). This directly impacts the accuracy of the target cell definition. Please provide an explanatory note in the text of M&M to clarify how this was controlled, thereby improving the reproducibility of the assay.

**********

what does this mean?). If published, this will include your full peer review and any attached files.

If you choose “no”, your identity will remain anonymous but your review may still be made public.

**Do you want your identity to be public for this peer review?**  For information about this choice, including consent withdrawal, please see our Privacy Policy

Reviewer #1: No

Reviewer #2: No

**********

[NOTE: If reviewer comments were submitted as an attachment file, they will be attached to this email and accessible via the submission site. Please log into your account, locate the manuscript record, and check for the action link "View Attachments". If this link does not appear, there are no attachment files.]

To ensure your figures meet our technical requirements, please review our figure guidelines: https://journals.plos.org/plosone/s/figures

You may also use PLOS’s free figure tool, NAAS, to help you prepare publication quality figures: https://journals.plos.org/plosone/s/figures#loc-tools-for-figure-preparation.

NAAS will assess whether your figures meet our technical requirements by comparing each figure against our figure specifications.

# Author comment: Author response to Decision Letter 2

**DOI:** 10.1371/journal.pone.0357999.r004

18 Aug 2026

Response to Reviewer 2

Minor comment

Figure 3D: I think that the Y-axis label contradicts the figure legend (line 383). The axis reads '% of Zombie Violet of CD56+ cells', whereas the legend defines the dead U251 cells as '% of Zombie Violet (+) of CD56(-) cells'. If it may be the case, please correct the axis label to accurately reflect the target population.

Additionally, U251 glioblastoma cells are of neuroectodermal origin and strongly express NCAM (CD56). This baseline expression can cause severe background staining or unexpected cross-reactivity depending on the CD56 antibody clone used to rule out effector cells (purified NKs). This directly impacts the accuracy of the target cell definition. Please provide an explanatory note in the text of M&M to clarify how this was controlled, thereby improving the reproducibility of the assay.

We apologize for the error in the Y-axis label of Figure 3D. The correct information is the one provided both in the figure legend and the paragraph titled “Cytotoxicity assays” in the Materials and Methods section. Both paragraphs explain that NK cytotoxicity was measured as the percentage of Zombie Violet-positive cells relative to the total number of CD56-negative cells. Therefore, as suggested by the reviewer, the Y-axis label in Figure 3D has been corrected to accurately reflect the target population.

Regarding the other point mentioned by the reviewer, we are aware that CD56 or NCAM (Neural Cell Adhesion Molecule) is a molecule that is not only strongly expressed in NK cells but it is also expressed in cells of neuroectodermal origin. For this reason, since U251 glioblastoma cells are larger and more granular than NK cells, our gating strategy was to first gate on tumor cells in an FSC vs. SSC dot plot. From this gate, we performed another dot plot of SSC vs. CD56 and selected the CD56-negative U251 cells from it. Finally, we determined the percentage of Zombie Violet-positive cells relative to the total number of CD56-negative U251 cells. To clarify this point, we included our gating strategy in the “Cytotoxicity assays” paragraph in the Materials and Methods section (Page 10, lines 226-229 of the revised version of the manuscript).

Attachment Submitted filename: Response_to_Reviewers_auresp_2.docx

# Editor report: Decision Letter 2

**DOI:** 10.1371/journal.pone.0357999.r005

**Authors:** Sriram Chitta

25 Aug 2026

Bacterial RNA downregulates MHC-I in tumor cell lines, promoting NK response and delaying tumor growth

PONE-D-26-11444R2

Dear Dr. Paula Barrionuevo

We’re pleased to inform you that your manuscript has been judged scientifically suitable for publication and will be formally accepted for publication once it meets all outstanding technical requirements.

Within one week, you’ll receive an e-mail detailing the required amendments. When these have been addressed, you’ll receive a formal acceptance letter and your manuscript will be scheduled for publication.

An invoice will be generated when your article is formally accepted. Please note, if your institution has a publishing partnership with PLOS and your article meets the relevant criteria, all or part of your publication costs will be covered. Please make sure your user information is up-to-date by logging into Editorial Manager at Editorial Manager® and clicking the ‘Update My Information' link at the top of the page. For questions related to billing, please contact billing support.

If your institution or institutions have a press office, please notify them about your upcoming paper to help maximize its impact. If they’ll be preparing press materials, please inform our press team as soon as possible -- no later than 48 hours after receiving the formal acceptance. Your manuscript will remain under strict press embargo until 2 pm Eastern Time on the date of publication. For more information, please contact onepress@plos.org.

Kind regards,

Sriram Chitta, Ph D

Academic Editor

PLOS One

Additional Editor Comments (optional):

Hi Dr Paula Barrionuevo

Congratulations! The manuscript titled "Bacterial RNA downregulates MHC-I in tumor cell lines, promoting NK response and delaying tumor growth" submitted to PLOS ONE is now completed review process. The research findings from your team are now ready for publication. Thank you for considering PLOS ONE to publish your research.

Best Regards

Sriram Chitta

AE

Reviewers' comments:

# Editor report: Acceptance letter

**DOI:** 10.1371/journal.pone.0357999.r006

**Authors:** Sriram Chitta

PONE-D-26-11444R2

PLOS One

Dear Dr. Barrionuevo,

I'm pleased to inform you that your manuscript has been deemed suitable for publication in PLOS One. Congratulations! Your manuscript is now being handed over to our production team.

At this stage, our production department will prepare your paper for publication. This includes ensuring the following:

* All references, tables, and figures are properly cited

* All relevant supporting information is included in the manuscript submission,

* There are no issues that prevent the paper from being properly typeset

You will receive further instructions from the production team, including instructions on how to review your proof when it is ready. Please keep in mind that we are working through a large volume of accepted articles, so please give us a few days to review your paper and let you know the next and final steps.

Lastly, if your institution or institutions have a press office, please let them know about your upcoming paper now to help maximize its impact. If they'll be preparing press materials, please inform our press team within the next 48 hours. Your manuscript will remain under strict press embargo until 2 pm Eastern Time on the date of publication. For more information, please contact onepress@plos.org.

You will receive an invoice from PLOS for your publication fee after your manuscript has reached the completed accept phase. If you receive an email requesting payment before acceptance or for any other service, this may be a phishing scheme. Learn how to identify phishing emails and protect your accounts at https://explore.plos.org/phishing.

If we can help with anything else, please email us at customercare@plos.org.

Thank you for submitting your work to PLOS One and supporting open access.

Kind regards,

PLOS One Editorial Office Staff

on behalf of

Dr. Sriram Chitta

Academic Editor

PLOS One
